# Long noncoding RNA: a dazzling dancer in tumor immune microenvironment

**DOI:** 10.1186/s13046-020-01727-3

**Published:** 2020-11-04

**Authors:** Yalu Zhang, Qiaofei Liu, Quan Liao

**Affiliations:** Department of General Surgery, Peking Union Medical College Hospital, Chinese Academy of Medical Sciences & Peking Union Medical College, Shuaifuyuan 1, Dongcheng District, 100730 Beijing, China

**Keywords:** LncRNA, Tumor immune microenvironment, Cancer immunotherapy

## Abstract

Long noncoding RNAs (lncRNAs) are a class of endogenous, non-protein coding RNAs that are highly linked to various cellular functions and pathological process. Emerging evidence indicates that lncRNAs participate in crosstalk between tumor and stroma, and reprogramming of tumor immune microenvironment (TIME). TIME possesses distinct populations of myeloid cells and lymphocytes to influence the immune escape of cancer, the response to immunotherapy, and the survival of patients. However, hitherto, a comprehensive review aiming at relationship between lncRNAs and TIME is missing. In this review, we focus on the functional roles and molecular mechanisms of lncRNAs within the TIME. Furthermore, we discussed the potential immunotherapeutic strategies based on lncRNAs and their limitations.

## Background

**Fig. 1:**
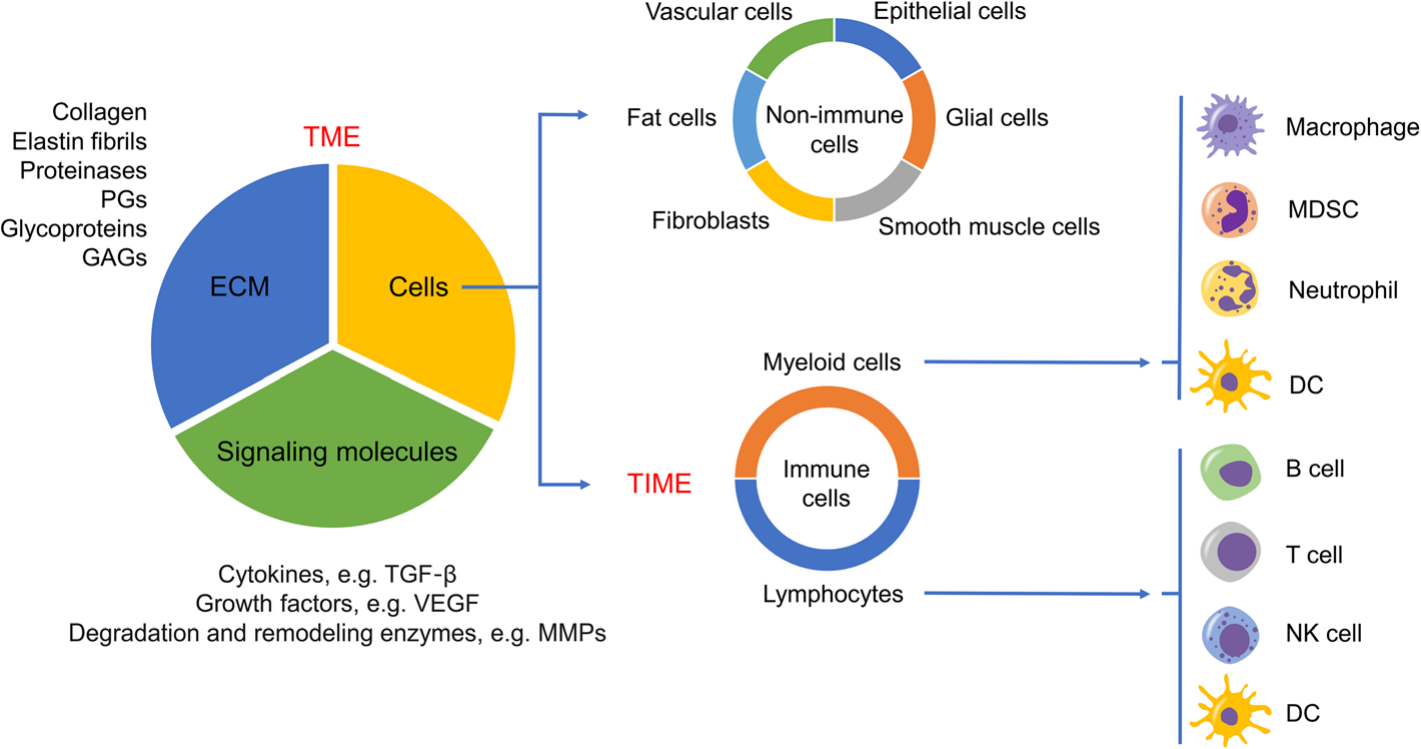
Schematic diagram of TME components. TME consists of cell component, the extracellular matrix (ECM) and abundant soluble signaling molecules. ECM is a macromolecular substance secreted by cells into the extracellular space and constitutes a complex network that supports tissue structure and the physiological activities of cells, including collagen, elastin fibrils, proteinases, proteoglycans (PGs), glycoproteins and glycosaminoglycans (GAGs). Signaling molecules in the TME include cytokines (e.g. TGF-β), growth factors (e.g. VEGF) and degradation and remodeling enzymes (e.g. MMPs). Substantial cells are divided into immune cells and non-immune cells. Non-immune cells are composed of epithelial, smooth muscle, vascular, glial, fat cells and fibroblasts. The infiltrating immune cells in the TME constitute the main body of TIME. TIME possesses distinct populations of myeloid cells and lymphocytes, two major categories of immune systems that act synergistically to initiate and reinforce innate and adaptive immunity in human, including macrophages, neutrophils, myeloid-derived suppressor cell (MDSC), B cells, T cells, natural killer (NK) cells, dendritic cells (DCs)

Cancer is not a chaotic malignant cell mass, but a delicate “hostile” organ, where many other cells are recruited and domesticated to become “accomplices”, thereby protecting themselves from recognition and attack by the immune system [1]. In addition to tumor cells, there are also important stromal components in tumor niche. The stroma is composed of substantial cells, including epithelial, fat, fibroblasts, smooth muscle, vascular, and immune cells along with the extracellular matrix (ECM) and abundant signaling molecules (Fig. 1) [2]. Together, they form the microenvironment in which the tumor is located, namely tumor microenvironment (TME). Besides, TME exhibits aberrant physiological conditions, like hypoxia, acidic extracellular pH and elevated interstitial fluid pressure, due to the malformed tumor vessels. The TME is an intricate physical and biochemical system that plays a significant role in tumor initiation, growth, distant metastasis, and affect the outcome of treatments [1, 2]. Among the components of TME, distinct populations of innate and adaptive immune cells consist of tumor immune microenvironment (TIME). TIME primarily consists of myeloid cells, lymphocytes and some other innate immune cells. Myeloid cells comprise macrophages, neutrophils, myeloid derived suppressor cells (MDSCs); lymphocytes include B cells, CD4^+^ T helper (Th) cells, regulatory T cells (Tregs), CD8^+^ cytotoxic T lymphocytes (CTLs); and the innate immune cells contain natural killer (NK) cells and dendritic cells (DCs) (Fig. 1). TIME influences the immune escape of cancer, the response to immunotherapy, and the survival rate of patients.

**Fig. 2:**
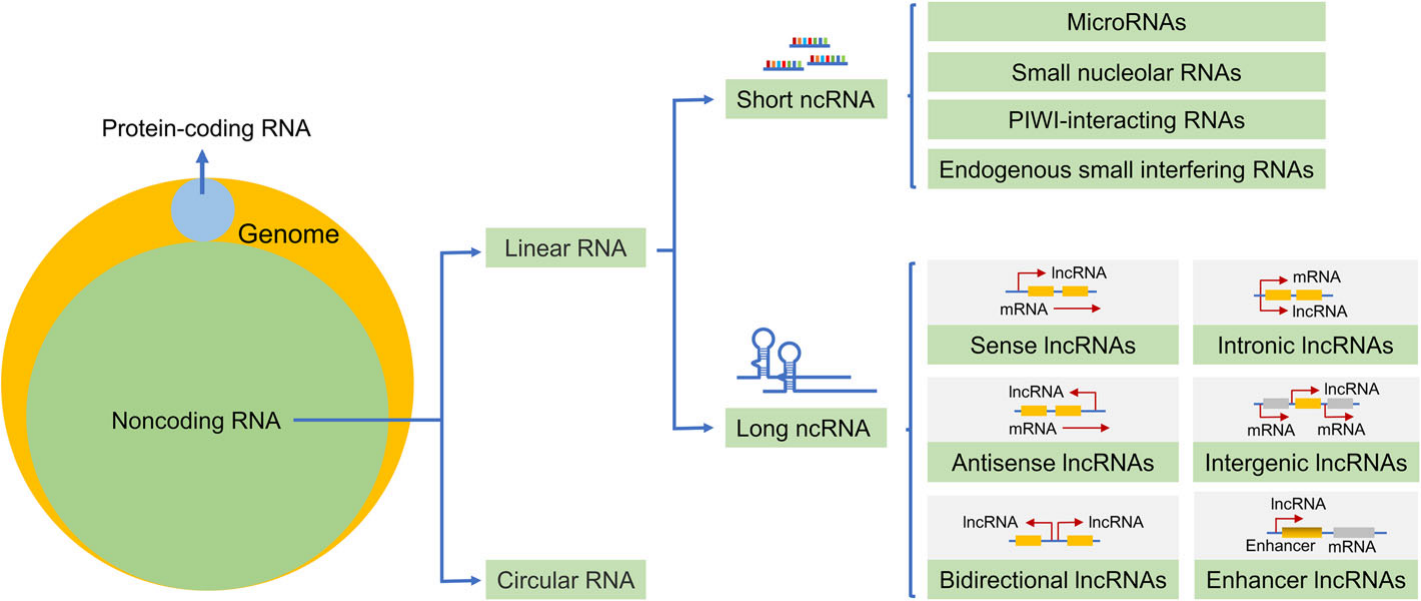
The proportion of ncRNAs in transcriptome and their classification. Protein-coding mRNAs only account for a small proportion in transcriptome, while the majority of them are non-coding RNAs (ncRNAs). Among them, ncRNAs can be divided into two main categories according to the molecular structure, linear RNA and circular RNA. The linear RNAs are further classified as small ncRNAs and long ncRNAs (lncRNAs) according to whether the length exceeds 200 nt. Short ncRNAs consist of microRNAs (miRNAs), small nucleolar RNAs, PIWI-interacting RNAs, endogenous small interfering RNAs. LncRNAs are composed of six types according to genomic location; sense, antisense, bidirectional, intronic, intergenic (lincRNAs) and enhancer

**Fig. 3:**
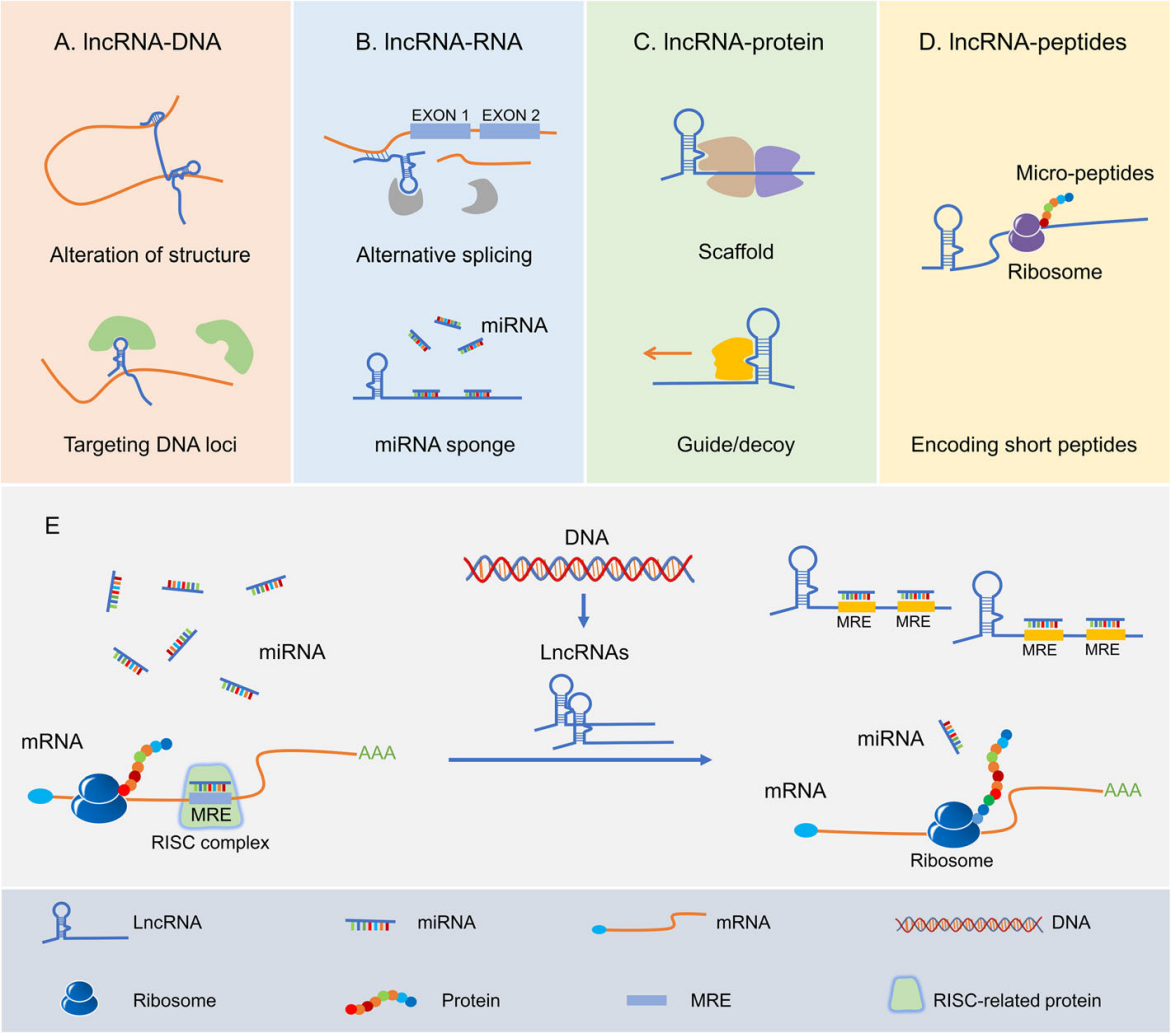
LncRNAs interactions and functions, and the mechanism of lncRNAs acting as miRNA sponge. **a** LncRNAs regulate gene expression by affecting local chromatin structure or recruiting regulatory proteins to specific loci. **b** LncRNAs facilitate RNA inhibition and degradation through interacting with mRNA and miRNA to control splicing or acting as a molecular sponge. **c** LncRNAs can serve as molecular scaffolds, guides, or decoys for regulatory proteins to regulate protein stability, activity, and localization. **d** Some lncRNAs are capable of encoding short peptides. **e** MiRNAs are able to directly bind to the matched regions of mRNAs by specific identification in a base-pairing manner, and thus inducing mRNA degradation at the post-transcriptional level by forming RNA-induced silencing complex (RISC) with related proteins such as Argonaute 2 (AGO2). LncRNAs harbor the miRNA response elements (MREs) with complementary miRNA binding sites and can competitively bind to miRNAs. Therefore, lncRNAs are capable of regulating the expression of mRNAs and exert its biological functions by sequestering functional miRNA molecules

In the human genome, about 93% of DNA can be transcribed into RNA, of which only 2% are protein-coding mRNAs, while the remaining 98% are called non-coding RNAs (ncRNAs) (Fig. 2) [3]. Among these ‘dark matters’, linear ncRNAs lack significant protein-coding potential and can be divided into two main categories according to whether the length exceeds 200 nucleotides (nt), namely short ncRNAs and long ncRNAs (lncRNAs) [4, 5]. The detailed classification is summarized in Fig. 2. Over the past two decades, a series of significant advances have been achieved in exploring the biogenesis, abundance, and function of lncRNAs across different species and cell types. Unlike miRNAs whose central function is to restrain mRNA translation by inducing degradation, lncRNAs can act as numerous roles to exert their functions by directly or indirectly interacting with DNA, RNA or protein, even can encode some short peptides (Fig. 3a-d) [6]. One of the most important mechanisms is to act as miRNA sponge and sequester miRNAs from target mRNAs, that is, to competitively bind miRNAs as competing endogenous RNA (ceRNA) of mRNAs, thereby regulating the levels of intracellular target mRNAs (Fig. 3e).

In particular, an increasing number of observations have proved that lncRNAs are involved in the regulation of substantial biological functions that determine cell fate and affect a series of physiological and pathological states [7], including cell apoptosis, differentiation, pyroptosis, autophagy and embryonic development [8]. More importantly, recent studies have also reported lncRNAs participate in cancer onset and progression via reprogramming the TIME [7, 9]. Indeed, ectopic expression of certain lncRNAs are potently associated with infiltration of immune cells and predict the prognosis of cancer patient. For instance, lncRNA-LINC00665 affects the infiltration level of macrophages and DCs, as well as suppresses Tregs and prevent T-cell exhaustion by acting as ceRNA to target FTX [10]. LncRNA-TCL6 is positively related to tumor-infiltrating lymphocytes (TILs) infiltration and immune checkpoint molecules, like PD-1, PD-L1, and CTLA-4 [11]. Oncogenic lncRNA LINK-A attenuates antigen presentation of tumor cells, which weakens immunosurveillance and facilitates cancer cells escape from immune checkpoints and survival of malignant cells [12]. Herein, we provide a general overview on the relationship between lncRNAs and TIME, and discuss immunotherapeutic potential of lncRNAs in cancer treatment.

### Tumor associated macrophages (TAMs)

Immune cells account for the remarkable percentage of the whole tumor tissue. Among them, macrophages, namely TAMs, stand for one of the most plentiful stromal compositions in the TIME [13]. It is generally recognized that TAMs derive from circulating bone marrow monocytes which infiltrate the primary and metastatic tumors due to being attracted by inflammatory cytokines. Afterwards, they differentiate into TAMs under the inductions of various signaling molecules in the TME [14]. However, recent evidence demonstrates that tissue resident macrophages derived from myeloid progenitors developed in fetal liver and yolk sac at the embryonic stage, where they are maintained via self-renewal [15–17]. TAMs are a major ingredient of the inflammatory cells infiltrate in diffusely distinct amounts in solid tumors [18]. They contribute to cancer progression through facilitating proliferation, metastasis and angiogenesis [19, 20]. Actually, TAMs are vital drivers of tumor-promoting inflammation, which recognized as the holistic factor that dampens the response to antitumor immunity and prompts cancer progression [18, 21]. Mechanistically, TAMs could remodel the structure of ECM, which facilitates the migration and invasion of the tumor cells by means of the TME and communicates with cancer cells or stromal cells through the secretion of cytokines, chemokines or growth factors [21].

Resting macrophages (M0) could be generally polarized into two major phenotypes according to specific microenvironment, classical pro-inflammatory activation (M1; IFN-γ/LPS-dependent) and alternative anti-inflammatory activation (M2; IL-4/IL-10/IL-13-dependent). Traditionally, macrophages have been deemed antitumorigenic when they release plentiful iNOS, TNF or MHC class II molecules and pro-tumorigenic when they secrete abundant IL-10, Arg1, CD163 or CD206 [22, 23]. However, accumulating evidence reveals that this kind of classification is oversimplification because the polarized macrophages can be further reprogrammed via reversing previous phenotype in respond to the alterative milieu [24]. Therefore, M1- and M2-polarized TAMs are only extremes of a continuum in a range of functional roles and the majority of the TAMs are variable states in the continuum between M1 and M2 [25].

Increasing data suggested that lncRNAs are crucial coordinators of changes in gene expression during macrophage polarization, although studies on lncRNAs in TAMs have just started [26]. LncRNAs are potently associated with macrophage recruitment, differentiation and activation [27]. In order to acquire differentially expressed lncRNAs profiles existing in different macrophage phenotypes, Huang et al. utilized microarray and bioinformatics methods and eventually identified 9343 and 4592 lncRNAs were deregulated in M (LPS + IFN-γ ) group and M (IL-4) group, respectively, in comparison to primary monocyte-derived macrophages [28]. Unsurprisingly, a recent study found that transcriptional factor STAT3 could facilitate the transcription of lncRNA RP11 − 389C8.2 (being named lnc-M2) and induce M2 macrophage polarization through the PKA/CREB pathway [29].

M2 polarization of macrophages is requisite for their function in immunologic tolerance, which might facilitate tumor progression. Plenty of pro-/anti-tumoral lncRNAs can help the realization of the process, that is, regulation of M1/M2 macrophage polarization via regulating the expression of downstream target proteins. LncRNA GNAS-AS1 promotes the growth and metastasis of estrogen receptor positive breast cancer through motivating M2 macrophage polarization via directly sponging miR-433-3p to target GATA3 [30]. Also, GNAS-AS1 can redirect polarization of macrophage in TME by miR-4319-mediated inhibition of NECAB3 [31]. Protein kinase C zeta (PKCζ) belongs to the PKC family and a key tumor suppressor gene. LncRNA-CCAT1 regulates macrophage polarization to prevent cell migration of prostate cancer by miR-148a/PKCζ axis [32]. Notch signaling is an important regulator in macrophage polarization [33–35]. LncRNA NIFK-AS1 could competitively bind to miR-146a to promote the expression of target gene Notch1, causing the inhibition of macrophage differentiation to M2 type in endometrial cancer [36]. LncRNA-XIST, subjected to the modulation of TCF-4, boosts M2 polarization of macrophages and is closely linked to tumor progression of lung cancer [37]. LncRNA-MM2P as a specific regulator of M2 polarization that acts by regulating phosphorylation of STAT6. Silence of lncRNA-MM2P alleviates macrophage-promoted angiogenesis and retards tumorigenesis [38]. Accumulation of lncRNA-ANCR in macrophages could fuel cancer metastasis through hindering M1 type differentiation via propelling FoxO1 ubiquitination degradation [39]. Down-regulated lincRNA-p21 promotes proinflammatory M1 polarization of macrophages in the TME, driven by MDM2 causing degradation of p53 and activation of the NF-κB/STAT3 pathway. In vivo, lincRNA-p21 knockdown macrophage adoptive transfer can hinder breast cancer progression [40]. Compared with M1 macrophages, the level of lncRNA cox-2 is lower in M2 macrophages. LncRNA cox-2 suppresses the tumor growth and immune evasion of hepatocellular carcinoma (HCC) via facilitating M1 polarization and curbing M2 polarization [41].

LncRNAs have the capability of regulating the protein secretion of TAMs to influence the survival and metastasis of tumor cells. In thyroid cancer, lncRNA MALAT1-mediated fibroblast growth factor-2 (FGF2) secretion from TAMs depresses release of inflammatory cytokines, induces vasculature formation and accelerates proliferation, invasion and migration of tumor cells [42]. Besides, TAMs can also influence the malignant behaviors of tumor cells by biological vectors rich in specific lncRNA. In pancreatic cancer, lncRNA SBF2-AS1 abundant in M2 macrophage-derived exosomes. Constrained SBF2-AS1 in these exosomes is beneficial to limit the tumorigenic capacity of neoplastic cells by elevating miR-122-5p expression and inhibiting expression of XIAP [43], a cytosolic suppressor of apoptosis-related protein [44].

More importantly, the polarization direction of TAMs is affected by tumor-secreted proteins, and this process is perhaps regulated by lncRNAs. A recent study indicated that upregulated LINC00662 contributed to secretion of WNT3A from cancer cells and activation of Wnt/β-catenin pathway in macrophages via paracrine manner, promotion of macrophages polarization to M2 type, causing worse prognosis of patients with cancer [45]. Silence of MALAT1 in HCC inhibits the production of VEGF-A, attenuates the angiogenesis, through restraining IL-10 secretion and the polarization of macrophage into M2 type, while stimulating the IL-6 secretion and the polarization of macrophage into M1 type [46]. Besides, macrophages can phagocytose and internalize tumor-derived exosomes, which are rich in lncRNAs with regulatory function and could induce macrophage polarization. In colorectal cancer, tumor-derived exosomes transport lncRNA-RPPH1 into macrophages to induce macrophage M2 polarization, thereby in turn propelling proliferation and metastasis of neoplastic cells [47]. In breast cancer, tumor cell-derived exosomes can accelerate M2 polarization and strengthen its cancer-promoting function through transmitting lncRNA-BCRT1 [48]. In HCC, tumor-derived exosomal lncRNA-TUC339 decreases pro-inflammatory cytokine production, alleviates co-stimulatory molecule expression, and compromises phagocytosis in environmental macrophages [49].

**Fig. 4:**
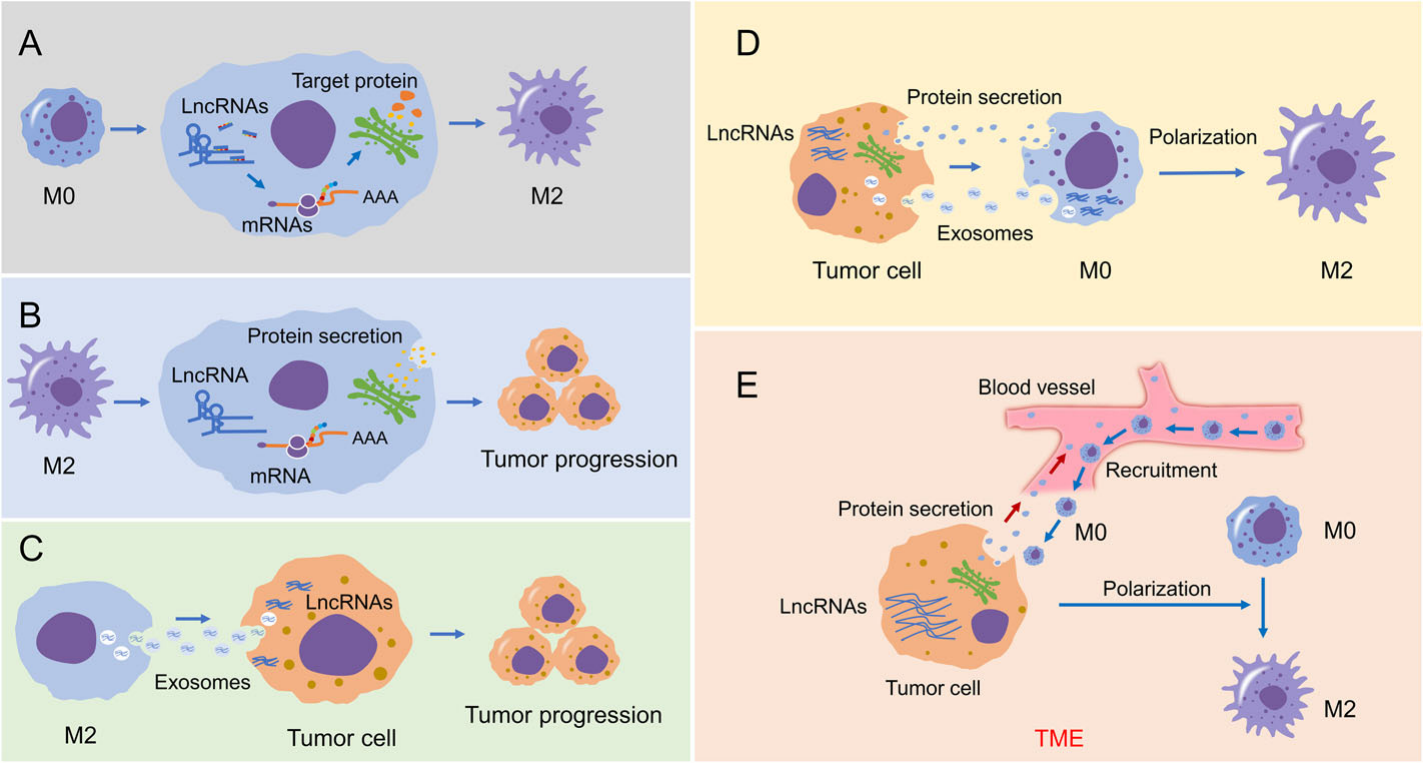
Role of lncRNAs in crosstalk between macrophages and tumor. **a** LncRNAs regulate M1/M2 macrophage polarization through miRNA-mediated alterations in the expression of downstream target proteins. **b** LncRNAs modulate the protein secretion of TAMs and affect the survival and metastasis of tumor cells. **c** TAMs can also influence the malignant behaviors of tumor cells by exosomes rich in specific lncRNA. **d** Macrophages phagocytose and internalize tumor-secreted proteins or tumor-derived exosomes rich in lncRNAs with regulatory function and thus induce macrophage polarization. **e** LncRNAs are involved in macrophage recruitment from circulating monocytes by regulating the production of secreted proteins, and in turn induce the polarization of macrophages into TAMs in the TME

In addition to regulating TAMs differentiation, lncRNAs are also involved in macrophage recruitment from circulating monocytes. Coagulation factor X (FX), a vitamin K-dependent plasma protein, possesses a potent chemotactic ability to recruit macrophages into the tumor tissue via binding to integrin αMβ2 on the macrophage surface and facilitates macrophage differentiation to the M2 subtype by potentiating the phosphorylation and activation of ERK1/2 and AKT [50]. The accumulation of lncRNA-CASC2c could suppress the expression and secretion of FX, thus exerting its immune regulatory role [50]. Interestingly, instead of acting as miR-338-3p ceRNA to augment FX expression, CASC2c interacts with and reciprocally inhibits miR-338-3p. Both CASC2c and miR-388-3p bind to FX and suppress its expression and secretion [50]. LncRNA CamK-A can facilitate the secretion of growth factors and cytokines derived from tumor cells to accelerate the transcription of multiple NF-κB downstream genes such as IL-6, VEGF etc., which result in angiogenesis, infiltrated macrophage recruitment and tumor microenvironment remodeling [51]. Taken together, these findings emphasize the importance of lncRNAs to regulate the recruitment, functions and polarization of macrophages, uncovering the potential significance of interactions between lncRNAs and TAMs, which are summarized in Table 1; Fig. 4.

**Table 1:** LncRNAs involved in TIME

**LncRNA**	**Related immune cell**	**Cancer type**	**Expression**	**Mediator miRNA**	**Target gene/pathway**	**Immunity-Related Mechanisms**	**Clinical significance**	**References**
LINK-A	—	TNBC	Upregulated in tumor cells	—	PtdIns(3,4,5)P3, GPCR, PKA, TRIM7	LINK-A-dependent downregulation of antigenicity and intrinsic tumor suppression by mediating the crosstalk between PtdIns(3,4,5)P3 and GPCR	Overall survival	[12]
LINC00665	—	HGSOC	Upregulated in tumor cells	—	FTX	Influencing the infiltration level of macrophages and DCs, and inhibiting Tregs and prevent T-cell exhaustion by FTX	Overall survival	[10]
GNAS-AS1	Macrophages	ER+ Breast cancer	Upregulated in tumor cells and M2 macrophages	miR-433-3p	GATA3	Accelerating M2 macrophage polarization	—	[30]
GNAS-AS1	Macrophages	Non-small cell lung cancer	Upregulated in tumor cells and M2 macrophages	miR-4319	NECAB3	Regulating macrophage polarization	Overall survival, metastasis-free survival	[31]
CCAT1	Macrophages	Prostate cancer	Downregulated in M2 macrophages	miR-148a	PKCζ	Regulating macrophage polarization	—	[32]
NIFK-AS1	Macrophages	Endometrial Cancer	Downregulated in M2 macrophages	miR-146a	Notch1	Inhibiting M2 macrophage polarization	—	[36]
XIST	Macrophages	Lung cancer	Upregulated in M2 macrophages	—	—	Regulating macrophage polarization	—	[37]
MM2P	Macrophages	—	Upregulated in M2 macrophages	—	STAT6	Accelerating M2 macrophage polarization	—	[38]
ANCR	Macrophages	Gastric cancer	Upregulated in tumor cells	—	FoxO1	Promoting tumor metastasis through hindering M1 type differentiation of macrophages by facilitating FoxO1 ubiquitination degradation	—	[39]
LincRNA-p21	Macrophages	Breast cancer	Upregulated in M2 macrophages	—	MDM2, P53, NF-κB, STAT3	Inhibiting macrophage polarization into pro-inflammatory M1 by promoting its interaction with p53	—	[40]
LncRNA-cox-2	Macrophages	HCC	Downregulated in M2 macrophages	—	—	Preventing immune evasion and metastasis of cancer by altering M1/M2 macrophage polarization	—	[41]
MALAT1	Macrophages	Thyroid cancer	Upregulated in tumor cells and M2 macrophages	—	FGF2	MALAT1-mediated FGF2 protein secretion from TAMs inhibits inflammatory cytokines release, promotes proliferation, migration, and invasion of tumor cells and induces vasculature formation	—	[42]
SBF2-AS1	Macrophages	Pancreatic cancer	Upregulated in M2 macrophages	miR-122-5p	XIAP	M2 macrophages secrete SBF2-AS1-rich exosomes to promote tumor progression.	—	[43]
LINC00662	Macrophages	HCC	Upregulated in tumor cells	miR-15a, miR-16, miR-107	WNT3A, Wnt/β-catenin signaling	Increasing tumor-derived WNT3A and activating Wnt/β-catenin signaling in macrophages via paracrine manner, and promoting M2 macrophages polarization	Overall survival, differentiation, tumor size, microvascular invasion	[45]
MALAT1	Macrophages	HCC	Upregulated in tumor cells	miR-140	VEGF-A	Regulating macrophage polarization	—	[46]
RPPH1	Macrophages	Colorectal cancer	Upregulated in tumor cells	—	TUBB3	Inhibiting TUBB3 ubiquitination and enhancing exosomes-mediated macrophages M2 polarization and influences the tumor microenvironment	Overall survival, TNM stages	[47]
BCRT1	Macrophages	Breast cancer	Upregulated in tumor cells and M2 [45]macrophages	miR-1303	PTBP3	Promoting M2 polarization of macrophages, mediated by exosomes, and accelerating cancer progression	Overall survival	[48]
TUC339	Macrophages	HCC	Upregulated in HCC-derived exosomes	—	—	Regulating macrophage activation and polarization	—	[49]
CASC2c	Macrophages	Glioblastoma	Downregulated in tumor cells	miR-338-3p	FX	Inhibiting macrophage migration and polarization to the M2 subtype by FX	—	[50]
CamK-A	Macrophages	Breast cancer	Upregulated in tumor cells	—	PNCK, IκBα, NF-κB, VEGF, IL-8, and GLUT3	Promoting infiltrated macrophage recruiting, angiogenesis, tumor microenvironment remodeling, and cancer development,	Overall survival, recurrence-free survival	[51]
Lnc-C/EBPβ	MDSCs	—	Upregulated in MDSCs	—	C/EBPβ isoform LIP	Controlling immunosuppressive function and differentiation of MDSCs by a set of target transcripts	—	[52]
Lnc-C/EBPβ	MDSCs	—	Upregulated	—	IL4il, C/EBPβ isoform LIP, WDR5	Promoting PMN-MDSCs but impede differentiation of M-MDSCs	—	[53]
RNCR3	MDSCs	—	Upregulated in MDSCs	miR-185-5p	Chop	Regulating MDSC differentiation and suppressive function to influence tumor growth by RNCR3/miR-185-5p/Chop axis	—	[54]
Lnc-chop	MDSCs	—	Upregulated in MDSCs	—	CHOP, C/EBPβ isoform LIP	Interacting with CHOP and the C/EBPβ isoform LIP to regulate immunosuppressive function of MDSCs	—	[55]
Olfr29-ps1	MDSCs	—	Upregulated	miR-214-3p	MyD88	Regulating the differentiation and function of M-MDSCs by a m6A-modified Olfr29-ps1/miR-214-3p/MyD88 regulatory network	—	[56]
Pvt1	MDSCs	—	Upregulated	—	C-myc	Regulating the immunosuppression activity of PMN-MDSCs by c-myc	—	[57]
AK036396	MDSCs	—	Upregulated	—	Ficolin B	Inhibiting maturation and accelerating immunosuppression of PMN-MDSCs by Enhancing the Stability of Ficolin B	—	[58]
MALAT1	MDSCs	Lung Cancer	Downregulated	—	—	Negatively regulating the generation of MDSCs	—	[59]
RUNXOR	MDSCs	Lung cancer	Upregulated	—	RUNX1	Mediating MDSCs associated immunosuppression by targeting RUNX1	Smoking history, TNM stage, histological tumor type and lymph node metastasis	[60]
HOTAIRM1	MDSCs	Lung Cancer	Downregulated	—	HOXA1	Regulating MDSCs associated immunosuppression by targeting HOXA1	Smoking history, TNM stage, histological tumor type and lymph node metastasis	[61]
HOTAIR	MDSCs	HCC	Upregulated	—	CCL2	Promoting proliferation of macrophages and MDSCs	—	[62]
Gm43181	Neutrophils	—	Upregulated in neutrophils	—	CXCR2	Provoking inflammation by regulating the recruitment and activation of neutrophils into the specific tissues	—	[63]
MALAT1	Neutrophils	—	Upregulated	—	p300, IL-8	Ameliorating the inflammatory injury by inhibiting chemotaxis of neutrophils through p300-mediated downregulation of IL-8	—	[64]
XIST	Neutrophils	—	Upregulated	miR-21	IL-12A	Increasing IL-12A by binding to miR-21, thereby inducing neutrophil extracellular trap formation	—	[65]
TCL6	Neutrophils	Breast cancer	Downregulated in tumor cells	—	—	—	Overall survival	[11]
HOTTIP	Neutrophils	Ovarian cancer	Upregulated in tumor cells	—	c-jun, IL-6, PD-L1	Enhancing IL-6 expression to potentiate immune escape of tumor cells by upregulating the expression of PD-L1 in neutrophils	Overall survival	[66]
Lnc-DC	DCs	—	Upregulated in DCs	—	STAT3, TLR9, TIMP, MMP	Regulating the differentiation and capacity of DCs by activating the transcription factor STAT3	—	[67–70]
NEAT1	DCs	—	Upregulated in DCs	miR-3076-3p	NLRP3	Inducing tolerogenic phenotype in DCs by inhibiting activation of NLRP3 inflammasome	—	[71]
HOTAIRM1	DCs	—	Downregulated in DCs	miR-3960	HOXA1	Regulating DC differentiation by competitively binding to endogenous miR-3960	—	[72]
Lnc-Dpf3	DCs	—	Upregulated in DCs	—	HIF-1α	CCR7-inducible lnc-Dpf3 restrains DC migration by inhibiting HIF-1α-mediated glycolysis	—	[73]
MALAT-1	DCs	Colon cancer	Upregulated in DCs	—	Snail	Blocking MALAT-1 significantly decreases the TADC-conditioned medium and CCL5-mediated migration and invasion by decreasing the Snail	—	[74]
SNHG16	B cells	DLBCL	Upregulated in tumor cells	miR-497-5p	PIM1	Promoting proliferation and inhibits apoptosis of lymphoma cells by targeting miR-497-5p/PIM1 axis	—	[75]
TUG1	B cells	DLBCL	Upregulated in tumor cells	—	MET	Regulating tumor growth by ubiquitination of MET	—	[76]
SNHG14	B cells	DLBCL	Upregulated in tumor cells	miR-5590-3p	ZEB1	SNHG14/miR-5590-3p/ZEB1 positive feedback loop promotes lymphoma progression and immune evasion by PD-1/PD-L1 checkpoint	—	[77]
MALAT1	B cells	DLBCL	Upregulated in tumor cells	miR-195	PD-L1	Promoting tumorigenesis and immune escape of lymphoma by sponging miR-195	—	[78]
MINCR	B cells	BL	Upregulated in tumor cells	—	AURKA, AURKB, and CDT1	Controlling cell cycle by participating in MYC transcriptional network	—	[79]
NEAT1	B cells	DLBCL	Upregulated in tumor cells	miR-34b-5p	GLI1	Promoting B cell proliferation and lymphomagenesis by the miR-34b-5p-GLI1 pathway.	—	[80]
FIRRE	B cells	DLBCL	Upregulated in tumor cells	—	Wnt/β-catenin pathway	Promoting the development of lymphoma by Wnt/β-catenin signaling pathway	Overall survival	[81]
PANDA	B cells	DLBCL	Downregulated in tumor cells	—	MAPK/ERK pathway	Inhibiting the growth of lymphoma by inactivation of MAPK/ERK signaling pathway	Overall survival, recurrence-free survival, B symptoms, Ann arbor stages, CHOP-like treatment, Rituximab and IPI	[82]
OR3A4	B cells	DLBCL	Upregulated in tumor cells	—	Wnt/β-catenin signaling	Promoting cell proliferation through activating Wnt/β-catenin signaling pathway	Overall survival	[83]
SMAD5-AS1	B cells	DLBCL	Downregulated in tumor cells	miR-135b-5p	APC, Wnt/β-catenin pathway	Inhibiting proliferation of lymphoma through Wnt/β-catenin pathway via targeting miR-135b-5p to elevate expression of APC	—	[84]
HULC	B cells	DLBCL	Upregulated in tumor cells	—	Bcl-2, cyclin D1	Regulating cell proliferation and inducing apoptosis by Bcl-2 and cyclin D1	Overall survival, progression–free survival, Ann Arbor stages, B symptoms, CHOP-like treatment, Rituximab and IPI	[85]
HOTAIR	B cells	DLBCL	Upregulated in tumor cells	—	PI3K/AKT/NF-κB pathway	Promoting cell proliferation by PI3K/AKT/NF-κB pathway	Overall survival, tumor size, clinical stage, B symptoms and IPI	[86]
LUNAR1	B cells	DLBCL	Upregulated in tumor cells	—	E2F1, cyclin D1 and p21	Regulating cell proliferation by E2F1, cyclin D1 and p21	Overall survival, progression-free survival, stage, rituximab and IPI	[87]
PEG10	B cells	DLBCL	Upregulated in tumor cells	—	—	—	Overall survival, B symptoms, CHOP-like treatment, Rituximab and IPI	[88]
MALAT-1	B cells	DLBCL	Upregulated in tumor cells	—	LC3-II/LC3-I, p62, ATG5	Regulating autophagy-related signaling pathway on chemotherapy resistance	—	[89]
FAS-AS1	B cells	Non-Hodgkin's lymphomas	Upregulated in tumor cells	—	RBM5	Regulating of the sFas expression by interacting with RBM5 to influence cell apoptosis	—	[90]
FENDRR	Tregs	HCC	Downregulated in Tregs	miR-423-5p	GADD45B	Inhibiting the Treg-mediated immune escape of tumor cells through upregulating GADD45B by sponging miR-423-5p	—	[91]
SNHG1	Tregs	Breast cancer	Upregulated in Tregs	miR-448	IDO	Accelerating the differentiation of Treg cells and promoting the immune escape of cancer by regulating miR-448/IDO axis	—	[92]
POU3F3	Tregs	Gastric cancer	Upregulated in Tregs	—	TGF-β/SMAD2/3 pathway	Promote the distribution of Tregs in peripheral blood T cell, increasing cell proliferation by recruiting TGF-β as well as activating TGF-β signal pathway	Tumor size	[93]
lnc-EGFR	Tregs	HCC	Upregulated in Tregs	—	EGFR, AP-1/NF-AT1 axis	Stimulating Treg differentiation, suppresses CTL activity and promoting HCC growth in an EGFR-dependent manner.	Tumor size	[94]
RP11-323N12.5	Tregs	Gastric cancer	Upregulated in tumor cells	—	YAP1, TAZ, TEAD, Hippo signaling	Promoting Treg cell differentiation by enhancing YAP1 transcription in T cells	Disease-free survival, stage	[95]
SNHG16	Tregs	Breast cancer	Upregulated in tumor cells	miR-16-5p	SMAD5	Breast cancer-derived exosomes transmit SNHG16 to induce CD73+γδ1 Treg cells by activation of the TGF-β1/SMAD5 pathway	—	[96]
Lnc-sox5	CTLs	Colorectal cancer	Upregulated in tumor cells	—	IDO1	Regulating the infiltration and cytotoxicity of CD3+CD8+T cells by IDO1, and unbalancing the TME	Metastasis	[97]
NEAT1	CTLs	Lung cancer	Upregulated in tumor cells	—	DNMT1, P53, cGAS/STING	Interacting with DNMT1 to regulate malignant phenotype of cancer cell and cytotoxic T cell infiltration by epigenetic inhibition of p53, cGAS, and STING	Tumor stage, lymph node metastasis.	[98]
NEAT1	CTLs	HCC	Upregulated in CTLs	miR-155	Tim-3	Regulating the antitumor activity of CD8+ T cells against HCC by miR-155/Tim-3 axis	—	[99]
Lnc-Tim3	CTLs	HCC	Upregulated in CTLs	—	Tim-3, Bat3, Lck/ NFAT1/AP-1 pathway	Exacerbating CD8+ T cell exhaustion by binding to Tim-3 and inducing nuclear translocation of Bat3	—	[100]
LINC00473	CTLs	Pancreatic cancer	Upregulated in tumor cells	miR-195-5p	PD-L1	Inhibiting activation of CD8+ T cells by sponging miR‐195‐5p to elevate the expression of PD‐L1	—	[101]
SNHG14	CTLs	DLBCL	Upregulated in tumor cells	miR-5590-3p	ZEB1, PD-1	Promoting apoptosis of CD8+ T cells by PD-1/PD-L1 immune checkpoint, and eventually leading to the immune evasion	—	[77]
MALAT1	CTLs	DLBCL	Upregulated in tumor cells	miR-195	PD-L1	Promoting apoptosis of CD8+ T cells and immune escape of lymphoma	—	[78]
GM16343	CTLs	—	Upregulated in CTLs	—	IFN-γ	Promoting IL-36β to regulate the TME by CD8+ T cells	Overall survival	[102]
NKILA	CTLs	breast and lung cancer	Upregulated in CTLs	—	NF-κB	Regulating T cell sensitivity to AICD by inhibiting NF-κB activity	Overall survival	[103]
INCR1	CTLs	—	Upregulated in CTLs	—	PD-L1, JAK2, STAT1, and IDO1, HNRNPH1	Regulating tumor IFNγ signaling and CTL-mediated killing	—	[104]
Lnc-CD56	NK cells	—	Upregulated in NK cells	—	CD56	Serving as a positive regulator of CD56 in primary human NK cells and differentiated NK cells from human CD34+ hematopoietic progenitor cells.	—	[105]
IFNG-AS1	NK cells	—	Upregulated in NK cells	—	IFNG	Triggering of the natural cytotoxicity receptors induces lncRNA IFNG-AS1 expression, and IFNG-AS1 increases IFN-γ secretion	—	[106]
GAS5	NK cells	Gastric cancer	Downregulated in NK cells	miR-18a	—	Promoting NK cell cytotoxicity against gastric cancer by regulating miR-18a	—	[107]
GAS5	NK cells	HCC	Downregulated in NK cells	miR-544	RUNX3	Enhancing the killing effect of NK cell on liver cancer by regulating miR-544/RUNX3	—	[108]
linc-EPHA6-1	NK cells	Lung cancer	—	miR-4485-5p	NKp46	IFNβ-induced exosomal linc-EPHA6-1 promotes cytotoxicity of NK cells by miR-4485-5p to increase NKp46 expression	—	[109]

*Abbreviations:AICD* Activation-induced cell death, *BL* Burkitt lymphoma, *CTLs* Cytotoxic T lymphocytes, *DCs* Dendritic cells, *DLBCL* Diffuse large B cell lymphoma, *HCC* Hepatocellular carcinoma, *HGSOC* High-grade serous ovarian cancer, *IPI* International prognostic index scores, *NK* Natural killer, *TNBC* Triple-negative breast cancer,* Tregs* Regulatory T cells

### MDSCs

The MDSCs are one of the cornerstones of the immunosuppressive shield and prevent the cancer from the patient’s immune system and immunotherapy. They are even vividly called the “queen bee” in the TIME [110]. As early as the late 1990s, it was found that a class of immune suppressive myeloid cells (CD11b^+^Gr-1^+^) in spleens of mice, and the phenotypically similar but functionally different from neutrophils and monocytes [111, 112]. Diverse phenotypic criteria were used to define this kind of cells in subsequent studies. Until 2007, the name MDSC, according to the origin and the functional feature, was proposed to unify various descriptions of these cells [113].

MDSCs comprise two main types of cells termed monocytic (M-MDSCs) and polymorphonuclear (PMN-MDSCs). M-MDSCs are morphologically and phenotypically like monocytes, and PMN-MDSCs are morphologically and phenotypically similar to neutrophils. Apart from above-mentioned two major cell communities, MDSCs contain a small fraction of cells with activity of myeloid colony formation such as myeloid progenitors and precursors [114]. In mice, M-MDSCs can be defined as CD11b^+^Ly6G^−^Ly6C^hi^ and PMN-MDSCs are described as CD11b^+^Ly6G^+^Ly6C^lo^. In humans, M-MDSCs are defined as CD11b^+^CD14^+^HLA-DR^−/lo^CD15^−^ and PMN-MDSCs as CD11b^+^CD14^−^CD15^+^ or CD11b^+^CD14^−^CD66b^+^ among peripheral blood mononuclear cells (PBMC) [115].

In the cancer setting, M-MDSCs are more dominant than PMN-MDSCs in terms of suppressive activity due to M-MDSCs could promptly mature into TAMs, despite PMN-MDSCs make up more than 80% of all MDSCs [116, 117]. More importantly, MDSCs refrain the immune response of T cells and mediate immunosuppression in tumor milieu via the expression of NOX2, NOS2 Arg-1, COX2, as well as production of NO and ROS [114]. Besides, MDSCs are able to facilitate the formation of Tregs and motivate fibroblasts differentiate into cancer-associated fibroblasts (CAFs) [118–120]. In addition to immune suppression, MDSCs also can secrete a series of cytokines, VEGF, MMP9, bFGF, etc., to influence angiogenesis and remodel the TIME [121, 122]. These result in the risk of dying from tumor is almost doubled in patients with MDSCs [123].

**Fig. 5:**
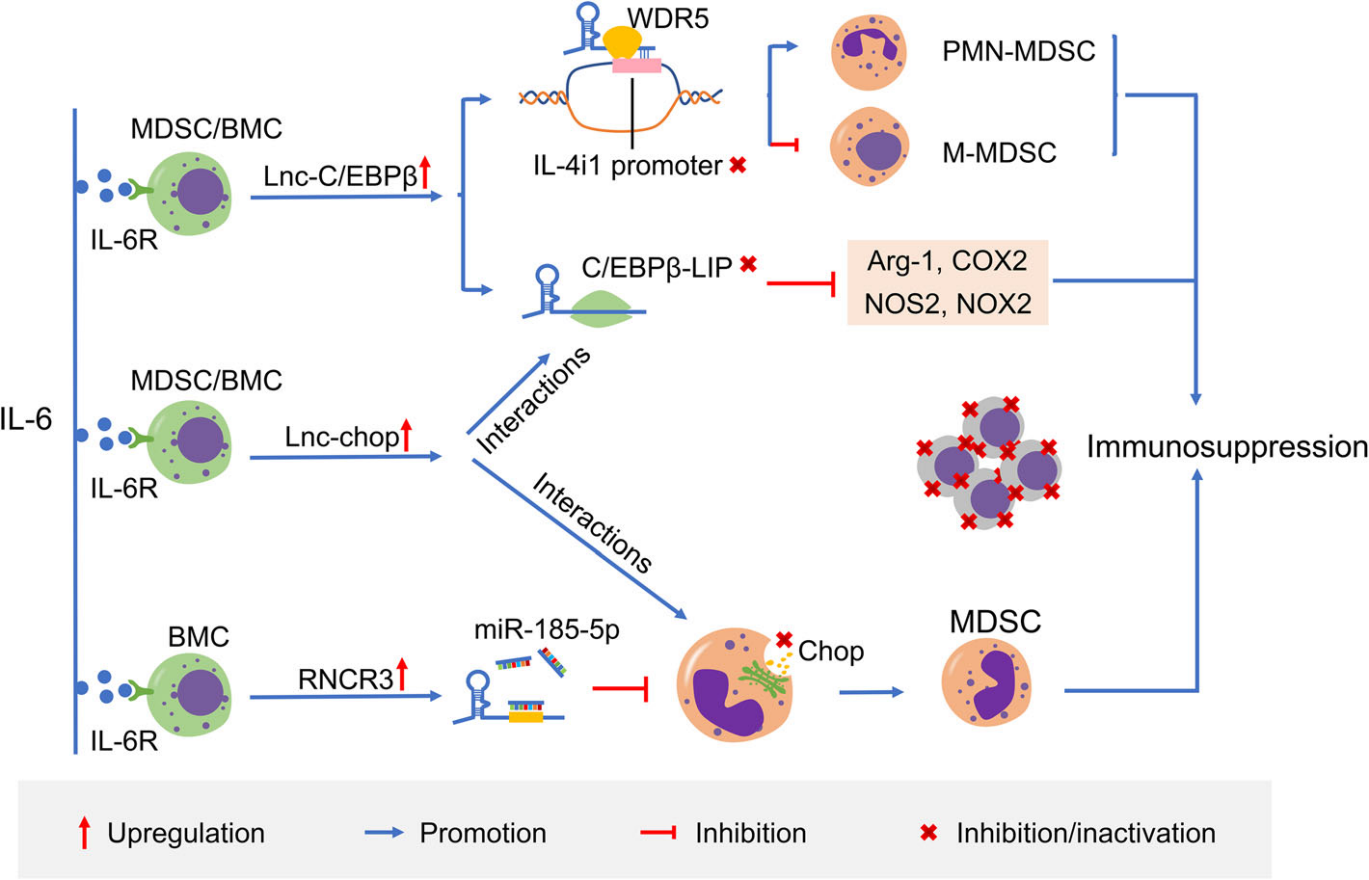
Schematic representation about the mechanisms of lnc-C/EBPβ, lnc-chop and RNCR3. BMC, bone marrow cell; MDSC, myeloid-derived suppressor cells; M-MDSC, monocytic MDSC; PMN-MDSC, polymorphonuclear MDSC; C/EBPβ-LIP, C/EBPβ isoform liver-enriched inhibitory protein (LIP); IL4i1, interleukin 4 induced gene-1

A number of studies have shown that lncRNAs are implicated in MDSCs differentiation and immunosuppressive function, and act as the crucial regulators. To date, the most of the experiments on MDSCs are performed on mice using murine cancer cells. In mice, transcription factors CCAAT/enhancer-binding protein (C/EBPβ) and C/EBP homologous protein (CHOP) pivotally regulate the expansion and function of MDSCs [124]. C/EBPβ has three isoforms and liver-enriched inhibitory protein (LIP) is one of the isoforms, which relies on forming heterodimers with other family members to manage gene expression due to lack of DNA activation domains [125]. There are three kinds of lncRNAs are identified in MDSCs; that is, lnc-C/EBPβ, lncRNA-RNCR3 and lnc-chop, which are significantly elevated in response to tumor-associated and extracellular inflammatory factors such as IL6. They are able to control function and differentiation of MDSCs in the TIME by regulating the downstream genes, C/EBPβ isoform LIP or/and CHOP (Fig. 5) [52, 54, 55]. Lnc-C/EBPβ binds the C/EBPβ isoform LIP to inhibit activation of C/EBPβ, affecting expansion of a suite of target transcripts including Arg-1, COX2, NOS2, NOX2 [52, 126]. Furthermore, lnc-C/EBPβ can facilitate differentiation of PMN-MDSCs but hinder differentiation of M-MDSCs via downregulating IL-4 induced gene 1 (IL4i1), an important gene mediating monocyte/macrophage differentiation [53, 127, 128]. Lnc-C/EBPβ also interacts with WDR5 to block the enrichment of H3K4me3 mark on the IL4i1 promoter region [53]. The conserved homo lnc-C/EBPβ has an analogous role with murine lnc-C/EBPβ. Human lnc-C/EBPβ may also exert a significant function in the differentiation and function of MDSCs in colorectal cancer patients [52, 53]. LncRNA-RNCR3 knockdown can stimulate Chop release and interrupt MDSC differentiation by binding to miR-185-5p, leading to reduction of the immunosuppressive activity of MDSCs and inhibition of tumor growth [54]. Lnc-chop directly interacts with both CHOP and the C/EBPβ isoform LIP to propel the activation of C/EBPβ and enrichment of H3K4me3, leading to expansion of a series of immunosuppression-related transcripts to govern differentiation and suppressive function of MDSCs [55].

The roles of Olfr29-ps1 depends on IL6-mediated *N*^*6*^-methyladenosine (m6A) modification, which not only increases Olfr29-ps1 expression, but also provokes the interaction between Olfr29-ps1 and miR-214-3p. miR-214-3p targets MyD88, which is crucial for the direct suppressive function of M-MDSCs [129, 130], to regulate the differentiation and development of M-MDSCs [56]. Apart from being related to M-MDSCs, lncRNAs are also involved in regulating the immunosuppressive activity of PMN-MDSCs. Under hypoxia environment, lncRNA plasmacytoma variant translocation 1 (Pvt1) could be enhanced by HIF-1α in PMN-MDSCs. Pvt1 silence can weaken PMN-MDSCs-mediated immunosuppression and recover antitumor T-cell responses, and accordingly retard tumor progression potentially targeting downstream c-myc [57]. The expression of Ficolin B could be considered as an indicator for PMN-MDSCs differentiation [131]. Through potentiating the stability of Ficolin B in a manner dependent on the ubiquitin-proteasome system, lncRNA-AK036396 dampens maturation and fosters immunosuppression of PMN-MDSCs [58].

In human lung cancer, lncRNA-MALAT1 is reduced in PBMCs from patients and negatively regulates the amount and proportion of MDSCs [59]. More importantly, both lncRNA-HOTAIRM1 and lncRNA-RUNXOR are tightly linked to the immunosuppression of MDSCs, but they have the opposite effect. Through analyzing the tumor tissues isolated from patients with lung cancer, it was found that HOTAIRM1 was negatively related to the level of Arg-1 and the proportion of MDSCs, while positively related to the ratio of Th1/CTL cells [61]. In contrast, lncRNA-RUNXOR level was positively linked to the level of Arg-1 and the proportion of MDSCs, whereas a negative linked to the ratio of Th1/CTL cells [60]. In addition, HOTAIRM1 could enhance HOXA1 level to abate the immunosuppression of MDSCs and reinforce the antitumor immune response [61], while RUNXOR facilitates the generation and suppressive activity of MDSCs by targeting RUNX [60]. In HCC, lncRNA-HOTAIR is probably implicated in the recruitment of macrophages and MDSCs to the TIME, since it can impulse expansion of macrophages and MDSCs via inducing secretion of CCL2 [62]. Collectively, these studies outline a critical role for lncRNAs in MDSC-mediated immunosuppression and might give us inspiration for immunotherapy (Table 1).

### Tumor associated neutrophils (TANs)

Accumulating evidence indicates TANs are involved in the pathogenesis of numerous cancers [132]. It has been reported that TANs possess diverse antitumor roles, including direct cytotoxicity towards cancer cells and repression of metastasis [132, 133]. On the contrary, another part of the studies indicated that TANs can fuel tumor growth and progression through stimulating the angiogenic switch, promoting neoplastic cell motility and invasion, and regulating other immune cells [134, 135]. TANs exhibit multifaceted and even opposing properties during tumor initiation and progression due to their heterogeneity and plasticity in the TIME [136]. In response to cytokines and inflammatory mediators released by neoplastic cells and cancer-associated cells, TANs can be polarized into divergent functional phenotypes to exert various pro- or antitumoral effects and alter cancer behavior [136].

The activation states of TANs polarization have been classified into an antitumorigenic phenotype N1 and a pro-tumorigenic phenotype N2 to mirror M1/M2 paradigm of macrophages [137]. Nevertheless, the classification of TANs in tumor has long been a matter of disputation because no specific surface marker has been identified to differentiate them, the most of data were obtained only from murine models and the lack of definite evidence in humans [135, 138]. With regard to TANs polarization, the binary N1/N2 classification is probably simplistic out of the analogous reasons that have been given against adopting M1 and M2 to sort TAMs [18, 139]. Similarly, TANs polarization might exist as a variable spectrum of phenotypes, instead of only two extremes.

**Fig. 6:**
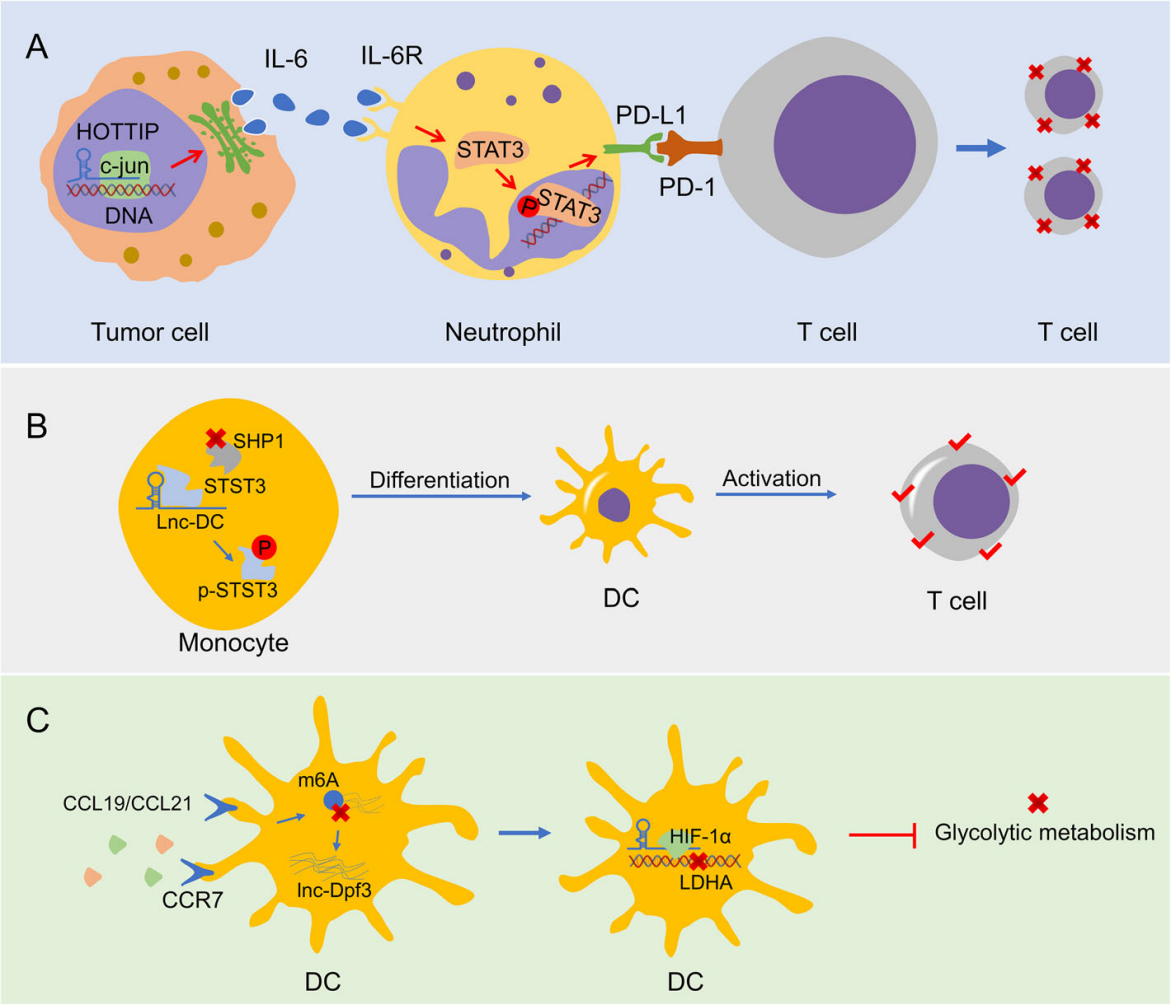
HOTTIP, lnc-DC and lnc-Dpf3 are involved in the regulation of immune cells. **a** HOTTIP in tumor cells by affecting IL-6 secretion to interact with neutrophils, increase the PDL-1 expression. **b** Lnc-DC influences DC differentiation and stimulate T cell activation by activating the transcription factor STAT3. **c** Lnc-Dpf3 affects the migratory ability of DCs by regulating their glucose metabolism

The short lifespan of neutrophils brings the technical difficulties to culture and handle these cells. As a consequence, there is a paucity in the literature hitherto studying the relationship between lncRNAs and neutrophils in the TME. Nevertheless, we still viewed the looming picture from the limited studies where lncRNA exerts a critical function in the pathogenesis of neutrophil-related diseases including inflammatory damage, transplantation and cancer, through affecting its quantities, phenotypes, expression and secretion of regulatory proteins. The C-X-C motif chemokine receptor 2 (CXCR2) belongs to the CXCR chemokine family and is expressed on the surface of neutrophils, where its activation could motivate neutrophil recruitment [140]. Resorting to reinforcing CXCR2 expression, lncRNA-Gm43181 can provoke inflammation through controlling the recruitment and activation of neutrophils into the specific tissues [63]. Quiescence of lncRNA-MALAT1 can ameliorate the inflammatory injury after lung transplant ischemia-reperfusion (LTIR) through depressing infiltration and activation of neutrophils and downregulating IL-8 [64]. LncRNA-XIST downregulation potentiates the apoptosis of polymorphonuclear neutrophils (PMNs) and dampens the formation of neutrophil extracellular trap (NET) via inhibition of IL-12A in rat model of lung transplantation [65]. In breast cancer, lncRNA-TCL6 correlates with neutrophils infiltration and manifests poor survival of cancer patients [11]. In ovarian cancer, upregulated lncRNA-HOTTIP can stimulate the production and secretion of IL-6 by regulating transcription factor c-jun, and increase the level of PD-L1 in TANs, and in turn refraining the activity of T cells, and ultimately facilitating immune escape of cancer cells (Fig. 6a) [66]. The evidence above indicates that lncRNAs may be able to influence tumor occurrence and development through regulating the function of neutrophils, and the regulatory relationship between lncRNAs and TANs remains a virgin place worth exploring (Table 1).

### DCs

Tumor-associated conventional DCs (cDCs) are considered to phagocytose debris from apoptotic cancer cells and transport cancer-related antigens to the draining lymph node where these antigens are presented to naïve CD4^+^ or CD8^+^ T cells and induce T cell priming and activation [141]. Although DCs equip with robust potential in anticancer immunity, the TME poses substantial challenges that often disturb normal DC functions to evade immune surveillance, and thus hinder the formation of protective immune responses [142]. Neoplastic cells can evolve a variety of mechanisms that allow them to thrive under adverse circumstance. Notably, multiple cytokines within the tumor milieu can directly influence the activity of infiltrating DCs to foster malignant progression. For example, IL-6, IL-10 and VEGF, generally overexpressed in the TIME can stimulate STAT3-related pathway to induce an immature and tolerogenic phenotype in tumor-associated dendritic cells (TADCs), thereby boosting cancer progression [143]. In fact, DC populations exhibiting immunosuppressive and tolerogenic properties are commonly found in the TIME of aggressive malignancies.

LncRNAs influence cellular function and differentiation via interacting with signaling molecules in the cytoplasm and modulating post-translational modification [67]. LncRNA lnc-DC (Gene symbol LOC645638) is exclusively expressed in human Lin^−^MHC-II^+^CD11c^+^ conventional DCs. Silence of lnc-DC refrains DC differentiation *in vitro* and *in vivo*, and decreases ability of DCs to stimulate T cell activation. Lnc-DC directly binds to STAT3 in the cytoplasm, stimulating STAT3 phosphorylation on tyrosine-705 via protecting STAT3 from binding to SHP1 and dephosphorylating it (Fig. 6b) [67]. On the basis of the similar mechanisms, some research groups reveal that lnc-DC motivates DC maturation and impedes trophoblast invasion without the involvement of CD4^+^ T cell, and the p-STAT3 inhibitor can restore the lnc-DC function by mediating the expression of MMP and TIMP [68]. Lnc-DC induces the excessive maturation of decidual DCs and the increase of Th1 cells in preeclampsia [69]. Also, lnc-DC modulates the HBV-induced immune response and cellular turnover via TLR9/STAT3 pathway in DCs [70].

Emerging evidence has indicated that lncRNAs are capable of regulating the infiltration, differentiation, metabolism of DCs, and affecting other immune cells including T cells to fine-tune the local immune milieu. LncRNA-NEAT1 induces tolerogenic phenotype in DCs by using the NLRP3 inflammasome as a molecular decoy for miR-3076-3p [71]. In the nucleus, miRNA let-7i regulates the expression of NEAT1 via interacting with transcription factor E2F1, which influences the distribution of H3K27ac in the promoter of NEAT1. In the mouse models, infusion with NEAT1-downregulated DCs reduce the infiltration of inflammatory cells, increase the quantities of Tregs, curb the proliferation of T cells [71]. These alterations inevitably induce immune tolerance. LncRNA-HOTAIRM1 plays a key role in myeloid development. HOTAIRM1 expression is decreased when monocytes differentiated into DCs [72]. Knockdown of HOTAIRM1 alters the level of some monocyte differentiation markers, like B7H2 and CD14. Besides, miR-3960 acts as competing endogenous RNA to regulate DC differentiate by targeting HOTAIRM1 and HOXA1, a repression gene of DC differentiation [72]. Lnc-Dpf3, induced by CCR7 chemokine receptor, inhibits DC migration via refraining HIF-1α-mediated glycolysis [73]. Concretely, CCR7 stimulation elevated lnc-Dpf3 by eliminating m^6^A modification to restrain RNA degradation. Lnc-Dpf3 knockdown facilitates CCR7-mediated DC migration, aggravating inflammatory degree and adaptive immune responses. Lnc-Dpf3 directly precludes HIF-1α-dependent transcription of the glycolytic gene LDHA, thereby retarding DC glycolytic metabolism and migration ability (Fig. 6c) [73]. Therefore, we speculate that lnc-Dpf3 reduction in DCs may be able to promote DC recruitment into the hypoxic TME through a similar mechanism, which in turn strengthens adaptive immune responses and improves the TIME. Tumor-infiltrating DCs secretes a large amount of CCL5 in human colon cancer specimens. Blocking CCL5 alleviates the promotion of tumor progression by TADCs [74]. Deficiency of lncRNA MALAT-1 strongly reduces CCL5-mediated invasion and migration via reducing Snail level [74]. These findings reveal that the MALAT-1/Snail signaling is essential for TADC-mediated tumor progression.

### B cells

Tumor-infiltrating B cells (TIBs) are present at all stages of tumor and exert crucial functions in shaping cancer advancement [144]. TIBs have been reported to the inconsistent roles in tumor immunity. On the one hand, it has been documented that B cells infiltrating plenty of cancers to generate class-switched affinity-matured antitumor antibodies *in situ* [145–147]. More than that, TIBs also promote T cell-mediated immune responses by acting as antigen-presenting cells to reinforce antitumor immunity [148]. On the other hand, TIBs also have been found to propel cancer progression, which have been designated as regulatory B cells (Bregs). In mouse and human studies, Bregs exhibit their immunosuppressive property by means of death ligands (TRAIL, FasL) or/and the secretion of anti-inflammatory factors (IL-10, TGFβ), which can impair NK and T cell responses and boost the pro-tumoral effects of Tregs, MDSCs, and TAMs, accordingly alleviating antitumor immune responses [148–150]. The homeostasis of B cells largely dependent on a range of factors with the TIME, such as intra-tumoral vascularity, inflammatory degree, cytokines, hypoxia, and cellular infiltration. Once that balance is broken, B cells might be polarized towards pro- or anti-tumor orientation.

Unfortunately, there are few studies concerning lncRNAs and B cells in the tumor milieu to date, and the majority of studies focus on B-cell related lymphomas triggered by dysfunction of lncRNAs during B cell development and maturation, including Burkitt lymphoma (BL), classical Hodgkin lymphoma (cHL) and diffuse large B cell lymphoma (DLBCL). Verma et al. identified 2632 novel lncRNAs in DLBCL by a systematic analysis from the poly-adenylated transcriptome of 116 primary DLBCL specimens, which expands the lymphoma transcriptome [151]. This indicating lncRNAs possibly participate in lymphoma regulation. LncRNA-SNHG16 facilitates proliferation and restrains apoptosis of lymphoma cells through regulating miR-497-5p-mediated expression of target gene PIM1 [75]. Depletion of lncRNA-TUG1 inhibits tumor growth through provoking ubiquitination of MET and subsequent degradation of MET *in vitro* and *in vivo* [76]. Several lncRNAs are capable of promoting immune evasion to alter tumor progression. LncRNA-SNHG14/miR-5590-3p/ZEB1 forms the positive feedback loop to foster immune escape and lymphoma progression via modulating PD-1/PD-L1 checkpoint [77]. LncRNA-MALAT1 fuels immune evasion of lymphoma by targeting miR-195 [78]. MALAT1 silence facilitates proliferation of CD8^+^ T cells and hinders EMT-like process by Ras/ERK signaling pathway [78].

More importantly, some of them are induced and regulated by MYC, a well-known transcription factor and is considered to be the major driving force in lymphoma development [152]. For example, lncRNA-MINCR acts as a regulator of the MYC transcriptional process to govern the expression of cell cycle genes, including AURKA, AURKB, and CDT1 [79]. MYC-induced lncRNA-NEAT1 facilitates lymphomagenesis and B cell proliferation by the miR-34b-5p/GLI1 axis [80]. MYC-activated lncRNA-FIRRE facilitates lymphoma progression through regulation of the nuclear translocation of β-catenin to activate Wnt/β-catenin pathway [81]. MYC is not the only modulator that interacts with the promoter region of lncRNA to promote its expression. P53-induced lncRNA-PANDA exerts the tumor-suppressive function in human lymphoma by inactivation of MAPK/ERK signaling pathway [82]. FOXM1-regulated upregulation of lncRNA-OR3A4 modulates cell apoptosis and proliferation through activating Wnt/β-catenin signaling pathway [83]. Apart from OR3A4 and FIRRE, LncRNA SMAD5-AS1 suppresses lymphoma growth also through the classic Wnt/β-catenin pathway, but dependent on the expression of APC [84].

In clinical terms, a variety of lncRNAs are associated with prognosis and affect clinical treatment in patients with B-cell related lymphoma. LncRNA HULC, HOTAIR, LUNAR1 and PEG10 can predict a poor clinical outcome, represent oncogenic activity, and the latter three lncRNAs have potential diagnostic value in DLBCL [85–88]. LncRNA MALAT-1 regulates the chemotherapy sensitivity of DLBCL by modulating autophagy-related proteins, including LC3-II/LC3-I, p62 and ATG5 [89]. Intriguingly, Su et al. [153] reported an artificially-designed i-lncRNA, targeting 13 oncogenic microRNAs according to complementarily sequences. The i-lncRNA curbed tumor growth *in vitro* and *in vivo* through effectively consuming a large number of oncogenic microRNAs mainly targeting a series of regulatory genes, such as PTEN, TIMP3, p38/MAPK, c-myc. In addition, soluble decoy receptor of Fas (sFas) can prevents Fas-ligand induced apoptosis. LncRNA FAS-AS1, regulated by EZH2-mediated histone methylation, is a novel regulator of the sFas expression through acting as a decoy for RBM5, to influence cell apoptosis in non-Hodgkin’s lymphomas (Table 1) [90].

### Tregs

At least two Treg subgroups have been generally identified in humans: those naturally developed within the thymus (natural; nTregs), and adaptive Tregs undergoing peripheral differentiation accommodate the environmental signals (induced; iTregs), such the gut, colon or placenta. The nTregs mediate suppression by cell contact-dependent mechanisms, including the Fas/FasL pathways and granzyme B/perforin, forming a major Treg subset for maintenance of peripheral immune tolerance [154]. The iTregs mediate suppression through contact-independent mechanisms resorting to the expression of immunosuppressive factors, such as TGF-β, IL-10, CTLA-4 and adenosine [155]. Understandably, tumor-infiltrating Tregs suppress the tumor-specific immune effector cells such as CD4^+^ T cells, cytotoxic CD8^+^ T cells, NK cells and DCs [156], and thus perturb the antitumor response of host. Besides, Tregs have been shown to stimulate angiogenesis and boost cancer aggressiveness [157, 158]. Clinically, Tregs dysfunction can evoke severe autoimmune diseases [159], but a hyperactive function of Tregs is also detrimental, because it dampens beneficial antipathogen and antitumor immunity [160]. Elevated Treg proportions of TILs, in particular decreased effector T cells, are strongly linked to poor prognosis in multiple cancers [161–163].

LncRNAs play a part in epigenetic control of Tregs fate. For example, lncRNA-Smad3 coordinate Smad3 locus accessibility to regulate iTreg polarization [164]. LncRNA-Flicr, a negative modulator of Foxp3, is specifically expressed in mature Tregs, and acts only in cis. It modifies chromatin accessibility in the CNS3/AR5 region of Foxp3 [165]. Hence, Flicr can significantly refrain antiviral responses and exacerbate autoimmune diseases via abating Treg activity [165]. Besides, foxp3 directly shapes the lncRNA transcriptome causing lncRNA transcriptome of Treg largely distinctive from that of non-regulatory CD4^+^ T cells [166]. Under normal physiological conditions, lncRNA-Flatr, a core member of the Treg lncRNA transcriptome, anticipates Foxp3 expression during Treg differentiation, indicating Flatr as part of the upstream cascade resulting in Treg differentiation [166].

Under the induction of cytokines or antigens in the local microenvironment, regulatory lncRNAs inside Tregs also show corresponding variation, promoting adaptive conversion of Tregs. LncRNA-FENDRR suppresses the Treg-mediated immune evasion of tumor cells by competitively bounding to miR-423-5p, which specifically targets DNA damage-related protein GADD45B. As a consequence, it potentiates tumorigenicity and cell growth in HCC [91]. Interference lncRNA-SNHG1 could repress Treg differentiation and in turn preclude the immune evasion of breast cancer by targeting miR-448 expression and diminishing IDO level [92]. Among aberrant expression profiling of lncRNAs in Tregs, lncRNA-POU3F3 possesses highest fold change and the most stable expression level. POU3F3 boosts the distribution of Tregs in peripheral blood T cell, resulting in the enhancement of proliferation in gastric cancer through recruiting TGF-β and activating TGF-β/SMAD2/3 pathway [93]. In HCC, lnc-EGFR promotes Treg differentiation, CTL inhibition and cancer progression in an EGFR-dependent manner. Functionally, lnc-EGFR specifically binds to EGFR to stabilize it by blocking c-CBL-mediated ubiquitination, resulting in downstream cascade activation [94].

**Fig. 7:**
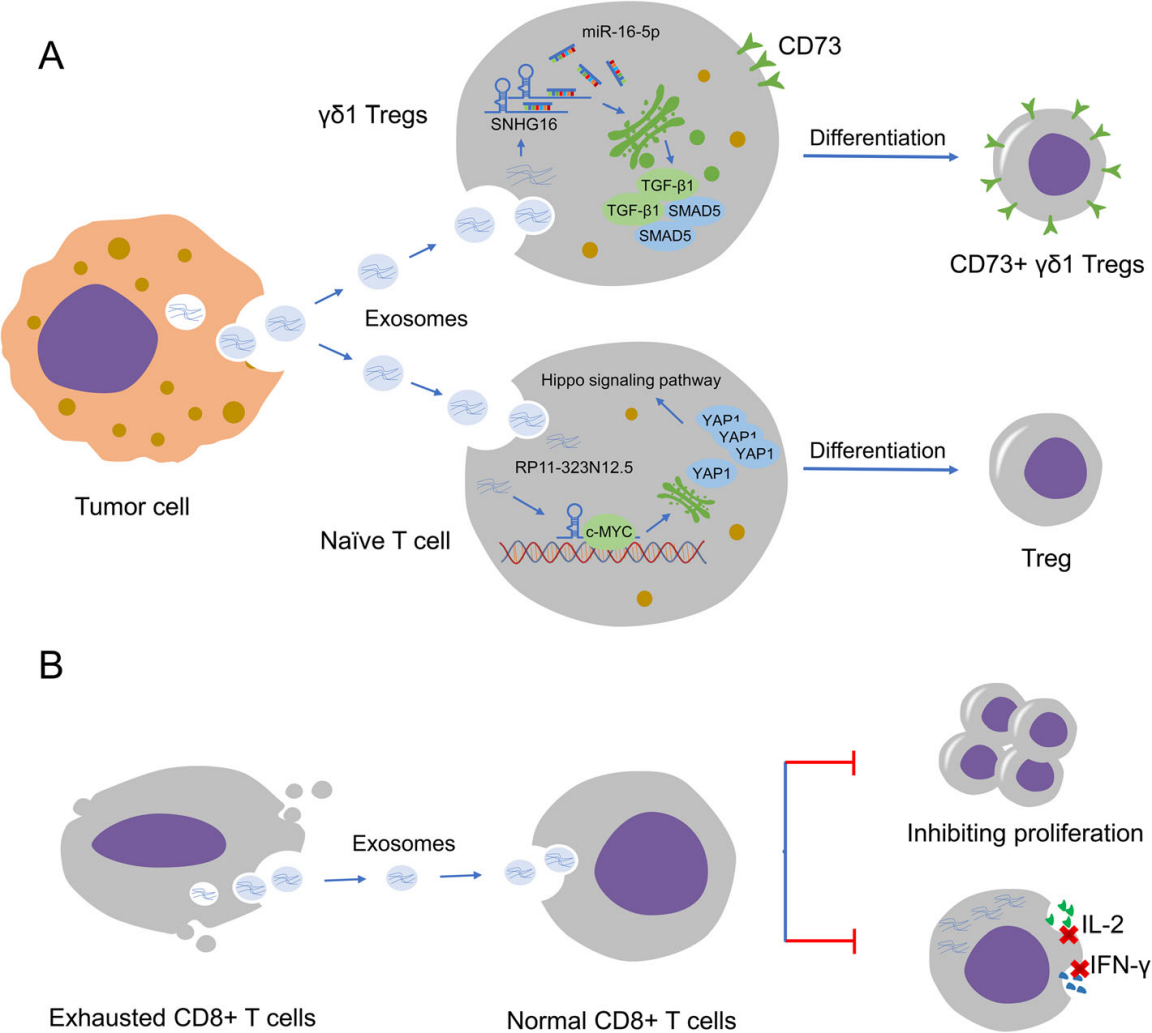
Schematic diagram of the mechanism of lncRNAs in T cells. **a** Exosomal SNHG16 and RP11-323N12.5 derived from tumor cell are internalized by T cells to affect their differentiation, and ultimately generates immunosuppressive effect. **b** Exosomes abundant in substantial lncRNAs are secreted by exhausted CD8^+^ T cells. These exosomes can be taken up by non-exhausted CD8^+^ T cells, and thus weakens their anticancer effect

Additionally, TILs can take up lncRNA-rich exosomes produced by neoplastic cells, prompting changes in lncRNAs within TILs, stimulating Tregs differentiation, and triggering immunosuppression and immune escape. Tumor cell secreting exosome containing abundant lncRNA RP11-323N12.5 acting on TILs to stimulate Treg cell differentiation dependent on YAP1 activation, an important factor in Hippo signaling pathways, ultimately promoting immunosuppression and tumor growth (Fig. 7a) [95]. Infiltrating CD73^+^γδ1Tregs exert a key immunosuppressive effect via adenosine generation in the breast TME [96]. Breast cancer-derived exosomal lncRNA SNHG16 stimulates the activation of the TGF-β1/SMAD5 pathway via mediating miR-16-5p and drives the conversion of γδ1 Tregs into the CD73^+^ immunosuppressive subtype (Fig. 7a; Table 1) [96].

### CTLs

A large number of lncRNA expression profiles have changed in mouse and human CD8^+^ T cells in response to viral infection [167]; of note, the most of lncRNAs expressed in CD8^+^ T cells harbor signatures of regulated promoters, secondary structures, and evolutionary conservation, indicating many of them are likely to play a part in fate decisions during adaptive immunity of antigen-driven differentiation [168]. Saadi et al.[169] adopted PMA/ionomycin to stimulate the P5424 cells, which partially simulated the β-selection process from pro-T cells like thymocytes, that was the CD4^−^CD8^−^ double negative (DN) to double positive (DP) transition, as well as aspects of succeeding T cell activation and maturation. Their research group uncovered a remarkable correlation between the dynamic expression of lncRNAs and neighbor coding genes including substantial new-found transcripts, thus revealing latent co-regulation. Furthermore, lncRNA Robnr displayed a significant role in terms of Bcl-2 induction, an important anti-apoptotic gene, and the concrete influence of the Robnr locus to leukemia progression and CTLs function and development [169].

The mechanism of lncRNAs in CD8^+^ T cells responding to antiviral processes may provide implications for the study of lncRNAs in the cancer setting. During the early stages of choriomeningitis virus infection, the transcription of the lncRNA-Morrbid is specifically induced by type I IFN stimulation and T cell receptor (TCR). As a response, the Morrbid and its locus govern the survival, proliferation and effector function of CD8^+^ T cell through controlling the pro-apoptotic factor, Bcl2l11, and activating the PI3K/Akt pathway [170]. LncRNA-CD160 eliminates the immune activity of CD8^+^ T cells via epigenetic mechanisms in hepatitis B virus infection. It can impair the secretion of TNF-α and IFN-γ in CD8^+^ T cells by recruiting histone-modification-related gene, HDAC11, to form a complex. This complex reinforces H3K9Me1 methylation and turns chromatin into the heterochromatin, thus the transcription of TNF-α and IFN-γ is retarded, inhibiting the immune response of CD8^+^ T cells [171].

Increasing studies have shown that lncRNA is the critical player for apoptosis, expansion, and cytotoxicity of CD8^+^ T cells in the TIME. To some extent, they determine the immune orientation by regulating CD8^+^ CLTs in the tumor milieu, whether it is anti-cancer or cancer-promoting. Lnc-sox5 regulates the infiltration and cytotoxicity of CD8^+^ T cells by indoleamine 2,3-dioxygenase 1 (IDO1), and unbalancing the TIME to affect tumorigenesis and advancement [97]. Reduction of lncRNA-NEAT1 can curb CD8^+^ T cell apoptosis and augment its cytolysis activity against cancer by binding to miR-155 targeting Tim-3, a key regulatory factor potentiating CD8^+^ T cell exhaustion [99]; moreover, through epigenetic suppression of p53/cGAS/STING pathway, NEAT1 directly interacting with DNMT1 to tune CD8^+^ T cells infiltration and T cell tumor-specific immune response, regulating malignant behavior of cancer cells [98]. Apart from NEAT1, lnc-Tim3 also can affect CTLs activity by Tim-3. Lnc-Tim3 aggravates CD8^+^ T cell exhaustion, a phenotype related to diminished antitumor immunity, through interacting with Tim-3 and inducing nuclear translocation of Bat3, and further enforcing p300-dependent p53 and RelA transcriptional activation of anti-apoptotic genes, like Bcl-2 and MDM2 [100]. Several studies have revealed that lncRNAs are able to regulate the apoptosis and expansion of CD8^+^ T cells through controlling the expression of PD-L1, and in turn escaping immune surveillance in the TIME. LINC00473 competitively binds to miR‐195‐5p to increase the expression of PD‐1 and PD‐L1 in cancer, thereby suppressing activation of CD8 ^+^ T cells and accelerating progression of pancreatic cancer [101]. Besides, above-mentioned SNHG14 also can induce interaction of tumor cells with CD8^+^ T cells by mediating miR-5590-3p, and causing apoptosis of CD8^+^ T cells via PD-1/PD-L1 immune checkpoint, and eventually contributing to the immune evasion of cancer cells [77]. LncRNA MALAT1 sponges miR-195 to regulate proliferation and apoptosis of CD8^+^ T cells by PD-L1, and in turn adjust immune escape abilities [78].

The research on mice found that difference of lncRNA-GM16343 was the most obvious between the groups by taking advantage of gene chip technology in mouse CD8^+^ T cells stimulated by IL-36β [102]. GM16343 stimulated the secretion of IFN-γ in CD8^+^ T cells, markedly reduced tumor volume of mice, and potently prolonged the survival time. It was indicated GM16343 profoundly dampened tumor growth through affecting antitumor immune function of CD8^+^ T cells (Table 1) [102]. Intriguingly, a recent study conducted by Wang et al. demonstrated that exosomes originated from exhausted CD8^+^ T cells could be internalized by non-exhausted CD8^+^ T cells, and impeding proliferation capacity and the secretion of cytokines, i.e. IFN-γ and IL-2, and thus attenuating the anticancer effect of normal CD8^+^ T cells (Fig. 7b) [172]. Importantly, they identified 257 candidate lncRNAs with different expression in exosomes of both. These lncRNAs actively implicated in tuning various biological processes of CD8^+^ T cell activity [172].

### NK cells

Through utilizing a suite of germline-encoded inhibitory and activating receptors, NK cells harbor the innate ability to rapidly recognize abnormal cells and respond to changes in ligand expression of tumor cells, that is, a tumor-associated profile stimulates activation of NK cells and triggers target cell killing [173]. Granzyme B and perforin, which can cause target cell apoptosis and osmotic lysis, are the key factors required for NK cell-mediated tumor killing via forming a synapse. The process can be marked by LAMP1 (also known as CD107a) expressed on the cell surface. Direct killing mode also occurs through death-receptor pathways such as FasL and TRAIL. In addition to cytotoxicity, NK cells also secrete pro-inflammatory cytokines, chemokines, e.g. IL-6, IL-13, IFN-γ, TNF, G-CSF and CCL5, which might exert direct antitumor activity [174]. It should be noted that NK cells are not only killers but also immunoregulatory cells that positive or negative effects against tumor responses through regulating T cells and DCs in multiple manner [175]. For example, NK cells are usually not a dominant lymphoid group in the TIME, but they can attract T cell infiltration and trigger immune responses by chemokine and cytokine secretion [175]. Clinically, emerging evidence suggests that NK cell activity has been negatively related to tumor incidence [176], and the mitigate NK cell function is potently correlated with worse prognosis [177].

During the differentiation of NK cells, a number of lncRNAs are involved in this progress. One study of NK-specific lncRNAs found that lncRNAs offer specific signatures to diverse NK populations, which may beneficial to different functions and phenotypes for NK cells from distinct cell compartments [105]. Among them, the expression of lnc-CD56, also called AB128931, is significantly upregulated in human NK cells and tightly associated with that of CD56, a canonical surface marker of NK cells and participates in NK cell development [178]. Lnc-CD56 serves as a positive modulator of CD56 in primary and differentiated NK cells from human CD34^+^ hematopoietic progenitor cells [105].Triggering of the natural cytotoxicity receptors induces lncRNA IFNG-AS1 expression, and that IFNG-AS1 increases IFN-γ secretion in human NK cells [106]. Therefore, IFNG-AS1 is a general modulator of the type I immune response.

LncRNA expression is intrinsically linked to NK-related cancer progression. LncRNA-GAS5 level is decreased in NK cells of cancer patients. The expression of GAS5 augment NK cell cytotoxicity, IFN-γ secretion, and the proportion of CD107a^+^ NK cells by sponging miR-544 to target RUNX3, and thus strengthening the killing effect of NK cells [108]. Furthermore, lncRNA GAS5 also potentiates the secretion of IFN-γ, TNF-α, as well as cytotoxicity of NK cells against gastric cancer via modulating miR-18a [107]. Notably, tumor-derived exosomes contain functional lncRNAs can be taken in NK cells for intercellular communication. IFNβ-induced exosomal linc-EPHA6-1 reinforces NK cell cytotoxicity against tumor cells and Zika virus infected tumor cells through miR-4485-5p-mediated the increase of NKp46, a vital natural cytotoxicity receptor [109]. Therefore, these data from aforementioned studies suggest that the considerable role of lncRNAs in regulation of NK cells infiltrating the tumor milieu is nonnegligible (Table 1).

### Immunotherapeutic potential of lncRNAs in the TIME

Plenty of lncRNAs or their fragments are stable and detectable in body fluids of patients with tumor, including the plasma, saliva and urine, as a non-invasion method [179]. They can predict cancer metastasis and the survival of patients, even the risk of recurrence. Therefore, lncRNAs may be the effective biomarkers for diagnosis and prognosis of cancer patients. The related content has been well-summarized and comprehensively discussed elsewhere [179–181]. Herein, we focus on the immunotherapeutic potential of lncRNAs in the TIME.

There are numerous advantages for lncRNA-related immunotherapy. Multiple regulatory sites of lncRNAs can interact with other molecules, which provides a broader prospect for the development of new structure-based anticancer drugs. As regulators, lncRNAs regulate an array of downstream target genes and participate in diverse cell signaling pathways, which makes lncRNAs own more powerful effectiveness in cancer therapy. Besides, a number of lncRNAs dysregulated in various tumors and possess cancer specificity. Subtype- or tissue-specific lncRNA expression is critical for development of new personalized treatments. The tumor-specific dysregulation and pivotal role of lncRNAs as oncogenes or tumor suppressor genes in regulating immune cell functions within the TIME has attracted increasing attention, arguing that lncRNAs are promising targets for immunotherapy in tumor. Restoration of aberrantly expressed vital lncRNAs by technical means fully mobilizes immune cell function, as well as evokes and enhances antitumor immune responses *in vivo* is an effective immunotherapeutic strategy.

### Available methods

**Fig. 8:**
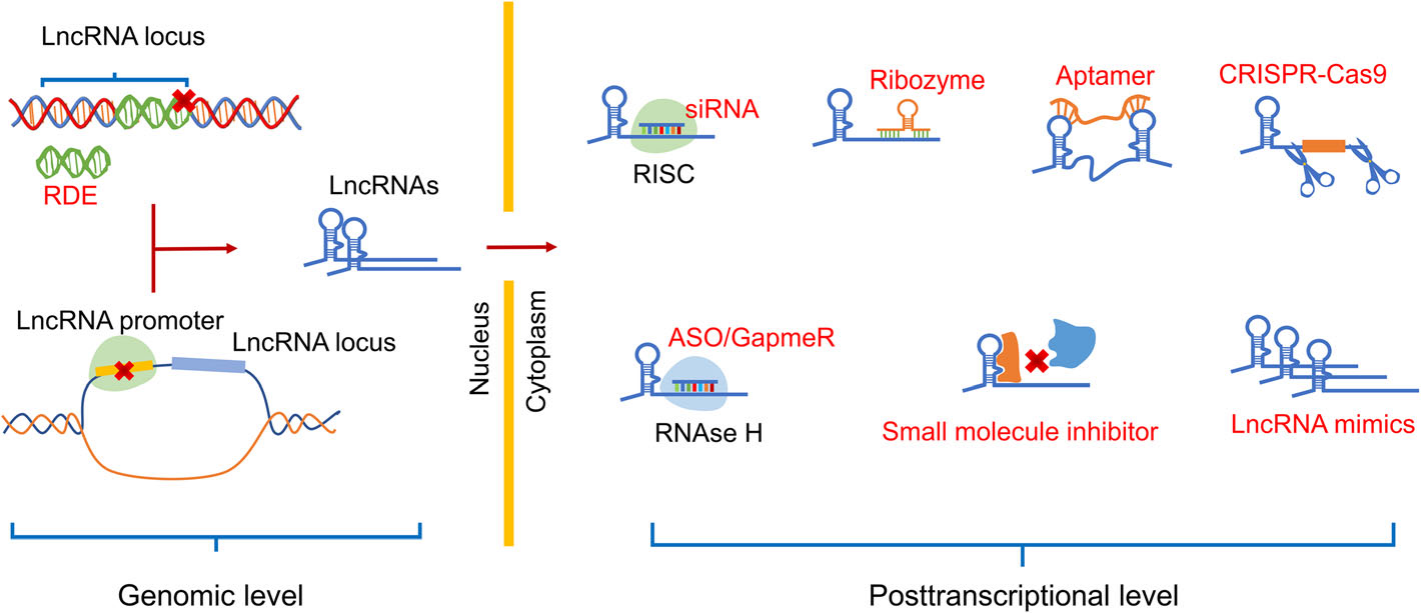
Schematic representation of available methods for regulating lncRNA levels in TIME

There are some approaches that may target lncRNA to regulate its expression in immune cells or tumors (Fig. 8) [4, 179]: (1) Integrating RNA destabilizing elements (RDEs) into the genome, a way to restrain lncRNAs via targeting specific genomic loci, produce an effect similar to knockout on gene expression, such as poly(A) signals that silence downstream sequences [182]. (2) Regulating lncRNA transcription via affecting the promoter activity of encoding lncRNA, such as inhibition of transcription factor binding to corresponding promoter. (3) LncRNA transcript destabilization or degradation, including lncRNA-specific siRNAs, antisense oligonucleotide (ASO), locked nucleic acid (LNA) GapmeRs, and ribozymes/deoxyribozmes. Similar to other genes, specific siRNAs form RNA-induced silencing complex (RISC) with related protein and bind to specific target sequence based on complementarity, causing degradation of target lncRNA [183]. ASOs are single stranded oligonucleotides with specific complementarity that can mediate the degradation of target lncRNAs using RNase H [184]. In terms of LNA GapmeRs, multiple single-stranded oligonucleotides consist of a DNA stretch flanked by LNA nucleotides and induce degradation of target lncRNA by RNAse H-dependent mechanism. Catalytic degradation is another effective way. Some enzymes, like ‘Hammerhead’-ribozymes, can target lncRNA and cleave it based on specific site in a protein-independent manner [185]. (4) Small molecule inhibitors that block interactions between lncRNAs and regulatory factors, i.e. DNA, RNA, protein or other interacting complexes, via specific binding site. (5) Aptamers can specifically bind to the structural regions of the target lncRNA and prevent it from binding to original partner, eliciting functional disruption of lncRNAs. (6) Gene editing to target lncRNAs, like CRISPR-Cas9, a major breakthrough in gene editing technology. Through inhibition (CRISPRi) or activation (CRISPRa), it is feasible to govern lncRNA expression in a transient or stable manner without altering the genomic sequence [186, 187]. (7) Synthetic lncRNA mimics. It is introduced into cells to increase the level of key lncRNAs [188].

### Strategies in combinational therapy

Over the past few years, several drugs, like PD-1, PDL-1 and CTLA-4 antibodies, have been developed for tumor immunotherapy with gratifying results. Nevertheless, the diverse resistance mechanisms of immunotherapy largely limit its effectiveness. Recently, an increasing number of studies suggested that lncRNAs play a vital role in drug resistance and immunotherapy resistance for cancers [12, 103, 104]. Accordingly, combining targeted lncRNA drugs with immunotherapy antibodies or chemotherapeutic medicine to maximize the efficacy of anticancer therapeutics may be the most effective strategy for cancer treatment.

Immunotherapy has revolutionized the treatment of malignancies especially through like immune checkpoint blockade (ICB) and chimeric antigen receptor T cell therapy (CAR-T) targeting distinct aspects of the immune-oncology cycle. The efficiency of ICB treatment primarily depends on T cell-recognized neoantigens exhibited by MHCs on cancer cells. It has been found that tumor-related lncRNAs mediates immune escape of tumor cells by suppressing antigen presentation. For instance, lncRNA LINK-A can influence MHC-1 stability of neoplastic cells. Treatment with LINK-A LNAs in combination with ICB can potently restrain tumor growth and increase the survival of the tumor-bearing mice, displaying synergistic efficacy between them [12]. The rationale for this therapeutic strategy is that inhibition of LINK-A expression by LINK-A LNAs restores the antigen presentation pathway of cancer cells, thereby improving the sensitivity of cancer to ICB treatment. Of note, treatment with LNA does not influence the distribution of immune cells, such as macrophages, MDSCs and CTLs [12]. It is, therefore, conceivable that combinatorial therapies concerning lncRNA ASOs or LNAs together with ICBs may show synergistic effects on antitumor immunity in humans.

Adoptive T cell therapies, especially CAR-T therapy, have achieved significant clinical effects in the treatment of hematological malignancies [189, 190]. Nevertheless, the efficacy of CAR-T therapy in solid malignant tumors is still limited. Recently, several studies have shown that lncRNAs acted as the auxiliary targets of CAR-T treatment. LncRNA-NKILA was reported to strongly increase T cell sensitivity to activation-induced cell death (AICD) by inhibiting NF-κB activity, and thus facilitating cancer cell immune evasion [103]. In the patient-derived xenograft (PDX) model, CD8^+^ T cells transfected with NKILA shRNA were implanted into immunocompromised mice, which effectively inhibited the tumor growth and overcame tumor immune evasion by increasing infiltration and cytotoxicity of CLTs and decreasing the AICDs [103]. These finding illuminating that engineering lncRNAs are able to improve the efficacy of adoptive T cell therapy for cancer. Analogously, Mineo et al. found that knockdown of lncRNA-INCR1 increased susceptibility of cancer cells to T cell-mediated killing *in vitro* and improved CAR-T cell efficacy *in vivo* via controlling tumor IFN-γ signaling [104]. Taken together, engineering lncRNAs in combination with CAR-T cells therapy may be a promising immunotherapeutic strategy.

On the other hand, the ideal combination immunotherapy should enhance effector cell function and reduce protector cell function [110]. Accordingly, simultaneously application of multiple lncRNAs for comprehensive treatment might be another sort of effective strategy, that is, elevation of antitumorigenic lncRNAs and reduction of pro-tumorigenic lncRNAs. We conceive that multi-method combination therapy and combined regulation of multiple immune cells (i.e., inhibition of infiltration and effects of MDSCs and enhancement of infiltration and cytotoxicity of CTLs) not only reduce the dose and side effects of each drug, but also increase the overall efficacy of immunotherapy.

### Current limitations of lncRNA immunotherapy

Although there are a variety of available methods and therapeutic strategies for lncRNA-related immunotherapy, there are still multiple limitations. Firstly, a major challenge is how to specifically deliver the respective molecules into targeted cells. To date, there are two main solutions for specific-target delivery. One of them is the utilization of artificial carriers, such as synthetic nanoparticles, to delivery biologically active constructs and lncRNAs [191]. Another is extracellular vesicles such as exosomes that mediate cell-to-cell communication and encapsulate a large number of key lncRNAs for successful targeted therapy [192]. The greatest advantage of using extracellular vesicles as delivery vehicles for transporting lncRNAs is that immune tolerance with the use of autologous vesicles and more effective tissue penetration into target tumor mass [4]. Components modified-nanoparticles and exosomes, based on the principle of ligand binding to receptor or antibody binding to ligand may improve the specificity of the delivery of lncRNAs into targeting immune cells. However, there are still some uncertainties, including whether they affect the other cellular components in TIME and cause safety problems after off-target. Secondly, lncRNAs are not simply single-stranded structures, and they also have secondary and even tertiary structures. This may result in the inability to intervene effectively, such as frequent disruption of efficient binding of these molecules to specific targets and difficulty in forming base pairs to induce their degradation as expected. Thirdly, unlike protein-coding genes, lncRNAs are poorly conserved among distinct species; thus, effective therapies developed through *in vitro* experiments and animal models may be difficult for human applications. Fourthly, there are still some other issues such as how to choose the proper lncRNA as drugs and how to ensure that lncRNAs can not cause unexpected side effects under the complex regulatory network.

So far, to our best knowledge, there are no clinical trials of lncRNAs for cancer treatment independently or in combination with other drugs. Studies on lncRNAs in cancer therapy still focus on the levels of molecular cytology and mouse tumor models. Therefore, our hitherto understanding of lncRNA mechanisms in TIME is only the tip of the iceberg. A series of intriguing questions remain unanswered.

## Conclusions

Collectively, we have summarized recent advancement involving of lncRNAs within the TIME, and their functions in the crosstalk between neoplastic cells and infiltrated immune cells, and the underlying molecular mechanisms. As well, we discussed potential immunotherapy strategies based on lncRNAs and their limitations. Undeniably, lncRNA molecules exert remarkable functions in remodeling TIME and regulating the immune escape of tumor cells. LncRNA-based targeted cancer immunotherapy is promising.

## Data Availability

Not applicable.

## Abbreviations

AICD: Activation-induced cell death
Arg-1: Arginase-1
ASO: Antisense oligonucleotide
BL: Burkitt lymphoma
Bregs: Regulatory B cells
C/EBPβ: CCAAT/enhancer-binding protein
CAFs: Cancer-associated fibroblasts
CAR-T: Chimeric antigen receptor T cell therapy
CCL2: Secretion of chemokine (C-C motif) ligand 2
cDCs: Conventional dendritic cells
ceRNA: Competing endogenous RNA
cHL: Classical Hodgkin lymphoma
CHOP: C/EBP homologous protein
COX2: Cyclooxygenase-2
CTLs: Cytotoxic T lymphocytes
CXCR2: C-X-C motif chemokine receptor 2
DCs: Dendritic cells
DLBCL: Diffuse large B cell lymphoma
ECM: Extracellular matrix
FGF2: Fibroblast growth factor-2
FX: Coagulation factor X
GADD45B: Growth arrest and DNA-damage-inducible beta protein
HCC: Hepatocellular carcinoma
HDAC11: Histone-modification enzyme gene histone deacetylase 11
ICB: Immune checkpoint blockade
IDO1: Indoleamine 2,3-dioxygenase 1
LAMP1: Lysosomal-associated membrane protein 1
LCMV: Lymphocytic choriomeningitis virus
LIP: Liver-enriched inhibitory protein
LNA: Locked nucleic acid
LncRNAs: Long noncoding RNAs
MDSCs: Myeloid derived suppressor cells
M-MDSCs: Monocytic MDSCs
NECAB3: N-terminal EF-hand calcium binding protein 3
NET: Neutrophil extracellular trap
NK cells: Natural killer cells
NO: Nitric oxide
NOS2: Nitric oxide synthase 2
NOX2: NADPH oxidase 2
PBMCs: Peripheral blood mononuclear cells
PDX: Patient-derived xenograft
PKCζ: Protein kinase C zeta
PMN-MDSCs: Polymorphonuclear MDSCs
PMNs: Polymorphonuclear neutrophils
Pvt1: Plasmacytoma variant translocation 1
RDEs: RNA destabilizing elements
RISC: RNA-induced silencing complex
ROS: Reactive oxygen species
TADCs: Tumor-associated dendritic cells
TAMs: Tumor associated macrophages
TANs: Tumor associated neutrophils
TCR: T cell receptor
Th cells: T helper cells
TIBs: Tumor-infiltrating B cells
TILs: Tumor-infiltrating lymphocytes
TIME: Tumor immune microenvironment
TME: Tumor microenvironment
TRAIL: TNF-related apoptosis-inducing ligand
Tregs: Regulatory T cells
XIAP: X‐linked inhibitor of apoptosis protein

## References

[1] Balkwill FR, Capasso M, Hagemann T (2012). The tumor microenvironment at a glance. J Cell Sci.

[2] Li HC, Fan XL, Houghton J (2007). Tumor microenvironment: The role of the tumor stroma in cancer. J Cell Biochem.

[3] Clark MB, Amaral PP, Schlesinger FJ, Dinger ME, Taft RJ, Rinn JL (2011). The reality of pervasive transcription. PLoS Biol.

[4] Parasramka MA, Maji S, Matsuda A, Yan IK, Patel T (2016). Long non-coding RNAs as novel targets for therapy in hepatocellular carcinoma. Pharmacol Therapeut.

[5] Ulitsky I, Bartel DP, lincRNAs (2013). Genomics, Evolution, and Mechanisms. Cell.

[6] Shi X, Sun M, Liu H, Yao Y, Song Y (2013). Long non-coding RNAs: a new frontier in the study of human diseases. Cancer Lett.

[7] Batista PJ, Chang HY (2013). Long Noncoding RNAs: Cellular Address Codes in Development and Disease. Cell.

[8] Wu H, Yang L, Chen LL (2017). The Diversity of Long Noncoding RNAs and Their Generation. Trends Genet.

[9] Huarte M, Rinn JL (2010). Large non-coding RNAs: missing links in cancer?. Hum Mol Genet.

[10] Wu M, Shang X, Sun Y, Wu J, Liu G (2020). Integrated analysis of lymphocyte infiltration-associated lncRNA for ovarian cancer via TCGA, GTEx and GEO datasets. PeerJ.

[11] Zhang Y, Li Z, Chen M, Chen H, Zhong Q, Liang L (2020). lncRNA TCL6 correlates with immune cell infiltration and indicates worse survival in breast cancer. Breast Cancer.

[12] Hu Q, Ye Y, Chan LC, Li Y, Liang K, Lin A (2019). Oncogenic lncRNA downregulates cancer cell antigen presentation and intrinsic tumor suppression. Nat Immunol.

[13] Li X, Liu R, Su X, Pan Y, Han X, Shao C (2019). Harnessing tumor-associated macrophages as aids for cancer immunotherapy. Mol Cancer.

[14] Liu Q, Liao Q, Zhao Y (2017). Chemotherapy and tumor microenvironment of pancreatic cancer. Cancer Cell Int.

[15] Zhu Y, Herndon JM, Sojka DK, Kim KW, Knolhoff BL, Zuo C (2017). Tissue-Resident Macrophages in Pancreatic Ductal Adenocarcinoma Originate from Embryonic Hematopoiesis and Promote Tumor Progression. Immunity.

[16] Biswas SK, Allavena P, Mantovani A (2013). Tumor-associated macrophages: functional diversity, clinical significance, and open questions. Semin Immunopathol.

[17] Chen Z, Feng X, Herting CJ, Garcia VA, Nie K, Pong WW (2017). Cellular and Molecular Identity of Tumor-Associated Macrophages in Glioblastoma. Cancer Res.

[18] Noy R, Pollard JW (2014). Tumor-associated macrophages: from mechanisms to therapy. Immunity.

[19] Qian BZ, Pollard JW (2010). Macrophage diversity enhances tumor progression and metastasis. Cell.

[20] De Palma M, Lewis CE (2013). Macrophage regulation of tumor responses to anticancer therapies. Cancer Cell.

[21] Mantovani A, Marchesi F, Malesci A, Laghi L, Allavena P (2017). Tumour-associated macrophages as treatment targets in oncology. Nat Rev Clin Oncol.

[22] Mantovani A, Sozzani S, Locati M, Allavena P, Sica A (2002). Macrophage polarization: tumor-associated macrophages as a paradigm for polarized M2 mononuclear phagocytes. Trends Immunol.

[23] DeNardo DG, Ruffell B (2019). Macrophages as regulators of tumour immunity and immunotherapy. Nat Rev Immunol.

[24] Miao X, Leng X, Zhang Q (2017). The Current State of Nanoparticle-Induced Macrophage Polarization and Reprogramming Research. Int J Mol Sci.

[25] Mantovani A, Allavena P (2015). The interaction of anticancer therapies with tumor-associated macrophages. J Exp Med.

[26] Recalcati S, Locati M, Marini A, Santambrogio P, Zaninotto F, De Pizzol M (2010). Differential regulation of iron homeostasis during human macrophage polarized activation. Eur J Immunol.

[27] Carpenter S, Aiello D, Atianand MK, Ricci EP, Gandhi P, Hall LL (2013). A long noncoding RNA mediates both activation and repression of immune response genes. Science.

[28] Huang Z, Luo Q, Yao F, Qing C, Ye J, Deng Y (2016). Identification of Differentially Expressed Long Non-coding RNAs in Polarized Macrophages. Sci Rep.

[29] Chen Y, Li H, Ding T, Li J, Zhang Y, Wang J (2020). Lnc-M2 controls M2 macrophage differentiation via the PKA/CREB pathway. Mol Immunol.

[30] Liu SQ, Zhou ZY, Dong X, Guo L, Zhang KJ (2020). LncRNA GNAS-AS1 facilitates ER + breast cancer cells progression by promoting M2 macrophage polarization via regulating miR-433-3p/GATA3 axis. Biosci Rep.

[31] Li Z, Feng C, Guo J, Hu X, Xie D (2020). GNAS-AS1/miR-4319/NECAB3 axis promotes migration and invasion of non-small cell lung cancer cells by altering macrophage polarization. Funct Integr Genomics.

[32] Liu J, Ding D, Jiang Z, Du T, Liu J, Kong Z (2019). Long non-coding RNA CCAT1/miR-148a/PKCzeta prevents cell migration of prostate cancer by altering macrophage polarization. Prostate.

[33] Pagie S, Gerard N, Charreau B (2018). Notch signaling triggered via the ligand DLL4 impedes M2 macrophage differentiation and promotes their apoptosis. Cell Commun Signal.

[34] Hans CP, Sharma N, Sen S, Zeng S, Dev R, Jiang Y (2019). Transcriptomics Analysis Reveals New Insights into the Roles of Notch1 Signaling on Macrophage Polarization. Sci Rep.

[35] Wang YC, He F, Feng F, Liu XW, Dong GY, Qin HY (2010). Notch signaling determines the M1 versus M2 polarization of macrophages in antitumor immune responses. Cancer Res.

[36] Zhou YX, Zhao W, Mao LW, Wang YL, Xia LQ, Cao M (2018). Long non-coding RNA NIFK-AS1 inhibits M2 polarization of macrophages in endometrial cancer through targeting miR-146a. Int J Biochem Cell Biol.

[37] Sun Y, Xu J (2019). TCF-4 Regulated lncRNA-XIST Promotes M2 Polarization Of Macrophages And Is Associated With Lung Cancer. Onco Targets Ther.

[38] Cao J, Dong R, Jiang L, Gong Y, Yuan M, You J (2019). LncRNA-MM2P Identified as a Modulator of Macrophage M2 Polarization. Cancer Immunol Res.

[39] Xie C, Guo Y, Lou S (2020). LncRNA ANCR Promotes Invasion and Migration of Gastric Cancer by Regulating FoxO1 Expression to Inhibit Macrophage M1 Polarization. Dig Dis Sci.

[40] Zhou L, Tian Y, Guo F, Yu B, Li J, Xu H (2020). LincRNA-p21 knockdown reversed tumor-associated macrophages function by promoting MDM2 to antagonize* p53 activation and alleviate breast cancer development. Cancer Immunol Immunother.

[41] Ye Y, Xu Y, Lai Y, He W, Li Y, Wang R (2018). Long non-coding RNA cox-2 prevents immune evasion and metastasis of hepatocellular carcinoma by altering M1/M2 macrophage polarization. J Cell Biochem.

[42] Huang JK, Ma L, Song WH, Lu BY, Huang YB, Dong HM (2017). LncRNA-MALAT1 Promotes Angiogenesis of Thyroid Cancer by Modulating Tumor-Associated Macrophage FGF2 Protein Secretion. J Cell Biochem.

[43] Yin Z, Zhou Y, Ma T, Chen S, Shi N, Zou Y (2020). Down-regulated lncRNA SBF2-AS1 in M2 macrophage-derived exosomes elevates miR-122-5p to restrict XIAP, thereby limiting pancreatic cancer development. J Cell Mol Med.

[44] Rehm M, Huber HJ, Dussmann H, Prehn JH (2006). Systems analysis of effector caspase activation and its control by X-linked inhibitor of apoptosis protein. EMBO J.

[45] Tian X, Wu Y, Yang Y, Wang J, Niu M, Gao S (2020). Long noncoding RNA LINC00662 promotes M2 macrophage polarization and hepatocellular carcinoma progression via activating Wnt/beta-catenin signaling. Mol Oncol.

[46] Hou ZH, Xu XW, Fu XY, Zhou LD, Liu SP, Tan DM (2020). Long non-coding RNA MALAT1 promotes angiogenesis and immunosuppressive properties of HCC cells by sponging miR-140. Am J Physiol Cell Physiol.

[47] Liang ZX, Liu HS, Wang FW, Xiong L, Zhou C, Hu T (2019). LncRNA RPPH1 promotes colorectal cancer metastasis by interacting with TUBB3 and by promoting exosomes-mediated macrophage M2 polarization. Cell Death Dis.

[48] Liang Y, Song X, Li Y, Chen B, Zhao W, Wang L (2020). LncRNA BCRT1 promotes breast cancer progression by targeting miR-1303/PTBP3 axis. Mol Cancer.

[49] Li X, Lei Y, Wu M, Li N (2018). Regulation of Macrophage Activation and Polarization by HCC-Derived Exosomal lncRNA TUC339. Int J Mol Sci.

[50] Zhang Y, Feng J, Fu H, Liu C, Yu Z, Sun Y (2018). Coagulation Factor X Regulated by CASC2c Recruited Macrophages and Induced M2 Polarization in Glioblastoma Multiforme. Front Immunol.

[51] Sang LJ, Ju HQ, Liu GP, Tian T, Ma GL, Lu YX (2018). LncRNA CamK-A Regulates Ca(2+)-Signaling-Mediated Tumor Microenvironment Remodeling. Mol Cell.

[52] Gao Y, Sun W, Shang W, Li Y, Zhang D, Wang T (2018). Lnc-C/EBPbeta Negatively Regulates the Suppressive Function of Myeloid-Derived Suppressor Cells. Cancer Immunol Res.

[53] Gao YH, Shang WC, Zhang D, Zhang SW, Zhang XP, Zhang Y (2019). Lnc-C/EBP beta Modulates Differentiation of MDSCs Through Downregulating IL4i1 With C/EBP beta LIP and WDR5. Front Immunol.

[54] Shang W, Tang Z, Gao Y, Qi H, Su X, Zhang Y (2017). LncRNA RNCR3 promotes Chop expression by sponging miR-185-5p during MDSC differentiation. Oncotarget.

[55] Gao Y, Wang T, Li Y, Zhang Y, Yang R (2018). Lnc-chop Promotes Immunosuppressive Function of Myeloid-Derived Suppressor Cells in Tumor and Inflammatory Environments. J Immunol.

[56] Shang W, Gao Y, Tang Z, Zhang Y, Yang R (2019). The Pseudogene Olfr29-ps1 Promotes the Suppressive Function and Differentiation of Monocytic MDSCs. Cancer Immunol Res.

[57] Zheng Y, Tian X, Wang T, Xia X, Cao F, Tian J (2019). Long noncoding RNA Pvt1 regulates the immunosuppression activity of granulocytic myeloid-derived suppressor cells in tumor-bearing mice. Mol Cancer.

[58] Tian X, Zheng Y, Yin K, Ma J, Tian J, Zhang Y (2020). LncRNA AK036396 Inhibits Maturation and Accelerates Immunosuppression of Polymorphonuclear Myeloid-Derived Suppressor Cells by Enhancing the Stability of Ficolin B. Cancer Immunol Res.

[59] Zhou Q, Tang X, Tian X, Tian J, Zhang Y, Ma J (2018). LncRNA MALAT1 negatively regulates MDSCs in patients with lung cancer. J Cancer.

[60] Tian X, Ma J, Wang T, Tian J, Zheng Y, Peng R (2018). Long non-coding RNA RUNXOR accelerates MDSC-mediated immunosuppression in lung cancer. Bmc Cancer.

[61] Tian X, Ma J, Wang T, Tian J, Zhang Y, Mao L (2018). Long Non-Coding RNA HOXA Transcript Antisense RNA Myeloid-Specific 1-HOXA1 Axis Downregulates the Immunosuppressive Activity of Myeloid-Derived Suppressor Cells in Lung Cancer. Front Immunol.

[62] Fujisaka Y, Iwata T, Tamai K, Nakamura M, Mochizuki M, Shibuya R (2018). et al. Long non-coding RNA HOTAIR up-regulates chemokine (C-C motif) ligand 2 and promotes proliferation of macrophages and myeloid-derived suppressor cells in hepatocellular carcinoma cell lines. Oncol Lett.

[63] Zhang NN, Zhang Y, Wang L, Xia JG, Liang ST, Wang Y (2019). Expression profiling analysis of long noncoding RNAs in a mouse model of ventilator-induced lung injury indicating potential roles in inflammation. J Cell Biochem.

[64] Wei L, Li J, Han Z, Chen Z, Zhang Q (2019). Silencing of lncRNA MALAT1 Prevents Inflammatory Injury after Lung Transplant Ischemia-Reperfusion by Downregulation of IL-8 via p300. Mol Ther Nucleic Acids.

[65] Li JW, Wei L, Han ZJ, Chen Z, Zhang Q (2020). Long non-coding RNA X-inactive specific transcript silencing ameliorates primary graft dysfunction following lung transplantation through microRNA-21-dependent mechanism. Ebiomedicine.

[66] Shang A, Wang W, Gu C, Chen C, Zeng B, Yang Y (2019). Long non-coding RNA HOTTIP enhances IL-6 expression to potentiate immune escape of ovarian cancer cells by upregulating the expression of PD-L1 in neutrophils. J Exp Clin Cancer Res.

[67] Wang P, Xue Y, Han Y, Lin L, Wu C, Xu S (2014). The STAT3-binding long noncoding RNA lnc-DC controls human dendritic cell differentiation. Science.

[68] Zhang W, Yang M, Yu L, Hu Y, Deng Y, Liu Y (2020). Long non-coding RNA lnc-DC in dendritic cells regulates trophoblast invasion via p-STAT3-mediated TIMP/MMP expression. Am J Reprod Immunol.

[69] Zhang W, Zhou Y, Ding Y. Lnc-DC mediates the over-maturation of decidual dendritic cells and induces the increase in Th1 cells in preeclampsia. Am J Reprod Immunol. 2017;77(6). 10.1111/aji.12647 28185352

[70] Zhuang L, Tian J, Zhang X, Wang H, Huang C (2018). Lnc-DC regulates cellular turnover and the HBV-induced immune response by TLR9/STAT3 signaling in dendritic cells. Cell Mol Biol Lett.

[71] Zhang M, Zheng Y, Sun Y, Li S, Chen L, Jin X (2019). Knockdown of NEAT1 induces tolerogenic phenotype in dendritic cells by inhibiting activation of NLRP3 inflammasome. Theranostics.

[72] Xin J, Li J, Feng Y, Wang L, Zhang Y, Yang R (2017). Downregulation of long noncoding RNA HOTAIRM1 promotes monocyte/dendritic cell differentiation through competitively binding to endogenous miR-3960. Onco Targets Ther.

[73] Liu J, Zhang X, Chen K, Cheng Y, Liu S, Xia M (2019). CCR7 Chemokine Receptor-Inducible lnc-Dpf3 Restrains Dendritic Cell Migration by Inhibiting HIF-1alpha-Mediated Glycolysis. Immunity.

[74] Kan JY, Wu DC, Yu FJ, Wu CY, Ho YW, Chiu YJ (2015). Chemokine (C-C Motif) Ligand 5 is Involved in Tumor-Associated Dendritic Cell-Mediated Colon Cancer Progression Through Non-Coding RNA MALAT-1. J Cell Physiol.

[75] Zhu Q, Li Y, Guo Y, Hu L, Xiao Z, Liu X (2019). Long non-coding RNA SNHG16 promotes proliferation and inhibits apoptosis of diffuse large B-cell lymphoma cells by targeting miR-497-5p/PIM1 axis. J Cell Mol Med.

[76] Cheng H, Yan Z, Wang X, Cao J, Chen W, Qi K (2019). Downregulation of long non-coding RNA TUG1 suppresses tumor growth by promoting ubiquitination of MET in diffuse large B-cell lymphoma. Mol Cell Biochem.

[77] Zhao L, Liu Y, Zhang J, Liu Y, Qi Q (2019). LncRNA SNHG14/miR-5590-3p/ZEB1 positive feedback loop promoted diffuse large B cell lymphoma progression and immune evasion through regulating PD-1/PD-L1 checkpoint. Cell Death Dis.

[78] Wang QM, Lian GY, Song Y, Huang YF, Gong Y (2019). LncRNA MALAT1 promotes tumorigenesis and immune escape of diffuse large B cell lymphoma by sponging miR-195. Life Sci.

[79] Doose G, Haake A, Bernhart SH, Lopez C, Duggimpudi S, Wojciech F (2015). MINCR is a MYC-induced lncRNA able to modulate MYC’s transcriptional network in Burkitt lymphoma cells. P Natl Acad Sci USA.

[80] Qian CS, Li LJ, Huang HW, Yang HF, Wu DP (2020). MYC-regulated lncRNA NEAT1 promotes B cell proliferation and lymphomagenesis via the miR-34b-5p-GLI1 pathway in diffuse large B-cell lymphoma. Cancer Cell Int.

[81] Shi X, Cui Z, Liu X, Wu S, Wu Y, Fang F (2019). LncRNA FIRRE is activated by MYC and promotes the development of diffuse large B-cell lymphoma via Wnt/beta-catenin signaling pathway. Biochem Biophys Res Commun.

[82] Wang Y, Zhang M, Xu H, Wang Y, Li Z, Chang Y (2017). Discovery and validation of the tumor-suppressive function of long noncoding RNA PANDA in human diffuse large B-cell lymphoma through the inactivation of MAPK/ERK signaling pathway. Oncotarget.

[83] Meng H, Zhao B, Wang Y (2020). FOXM1-induced upregulation of lncRNA OR3A4 promotes the progression of diffuse large B-cell lymphoma via Wnt/beta-catenin signaling pathway. Exp Mol Pathol.

[84] Zhao CC, Jiao Y, Zhang YY, Ning J, Zhang YR, Xu J (2019). Lnc SMAD5-AS1 as ceRNA inhibit proliferation of diffuse large B cell lymphoma via Wnt/beta-catenin pathway by sponging miR-135b-5p to elevate expression of APC. Cell Death Dis.

[85] Peng W, Wu J, Feng J (2016). Long noncoding RNA HULC predicts poor clinical outcome and represents pro-oncogenic activity in diffuse large B-cell lymphoma. Biomed Pharmacother.

[86] Yan Y, Han J, Li Z, Yang H, Sui Y, Wang M (2016). Elevated RNA expression of long noncoding HOTAIR promotes cell proliferation and predicts a poor prognosis in patients with diffuse large B cell lymphoma. Mol Med Rep.

[87] Peng W, Feng J (2016). Long noncoding RNA LUNAR1 associates with cell proliferation and predicts a poor prognosis in diffuse large B-cell lymphoma. Biomed Pharmacother.

[88] Peng W, Fan H, Wu G, Wu J, Feng J (2016). Upregulation of long noncoding RNA PEG10 associates with poor prognosis in diffuse large B cell lymphoma with facilitating tumorigenicity. Clin Exp Med.

[89] Li LJ, Chai Y, Guo XJ, Chu SL, Zhang LS (2017). The effects of the long non-coding RNA MALAT-1 regulated autophagy-related signaling pathway on chemotherapy resistance in diffuse large B-cell lymphoma. Biomed Pharmacother.

[90] Sehgal L, Mathur R, Braun FK, Wise JF, Berkova Z, Neelapu S (2014). FAS-antisense 1 lncRNA and production of soluble versus membrane Fas in B-cell lymphoma. Leukemia.

[91] Yu Z, Zhao H, Feng X, Li H, Qiu C, Yi X (2019). Long Non-coding RNA FENDRR Acts as a miR-423-5p Sponge to Suppress the Treg-Mediated Immune Escape of Hepatocellular Carcinoma Cells. Mol Ther Nucleic Acids.

[92] Pei X, Wang X, Li H (2018). LncRNA SNHG1 regulates the differentiation of Treg cells and affects the immune escape of breast cancer via regulating miR-448/IDO. Int J Biol Macromol.

[93] Xiong G, Yang L, Chen Y, Fan Z (2015). Linc-POU3F3 promotes cell proliferation in gastric cancer via increasing T-reg distribution. Am J Transl Res.

[94] Jiang RQ, Tang JW, Chen Y, Deng L, Ji J, Xie Y (2017). The long noncoding RNA lnc-EGFR stimulates T-regulatory cells differentiation thus promoting hepatocellular carcinoma immune evasion. Nat Commun.

[95] Wang J, Huang F, Shi Y, Zhang Q, Xu S, Yao Y (2020). RP11-323N12.5 promotes the malignancy and immunosuppression of human gastric cancer by increasing YAP1 transcription. Gastric Cancer.

[96] Ni C, Fang QQ, Chen WZ, Jiang JX, Jiang Z, Ye J (2020). Breast cancer-derived exosomes transmit lncRNA SNHG16 to induce CD73 + gammadelta1 Treg cells. Signal Transduct Target Ther.

[97] Wu K, Zhao Z, Liu K, Zhang J, Li G, Wang L (2017). Long noncoding RNA lnc-sox5 modulates CRC tumorigenesis by unbalancing tumor microenvironment. Cell Cycle.

[98] Ma F, Lei YY, Ding MG, Luo LH, Xie YC, Liu XL (2020). LncRNA NEAT1 Interacted With DNMT1 to Regulate Malignant Phenotype of Cancer Cell and Cytotoxic T Cell Infiltration via Epigenetic Inhibition of p53, cGAS, and STING in Lung Cancer. Front Genet.

[99] Yan K, Fu Y, Zhu N, Wang Z, Hong JL, Li Y (2019). Repression of lncRNA NEAT1 enhances the antitumor activity of CD8(+)T cells against hepatocellular carcinoma via regulating miR-155/Tim-3. Int J Biochem Cell Biol.

[100] Ji J, Yin Y, Ju H, Xu X, Liu W, Fu Q (2018). Long non-coding RNA Lnc-Tim3 exacerbates CD8 T cell exhaustion via binding to Tim-3 and inducing nuclear translocation of Bat3 in HCC. Cell Death Dis.

[101] Zhou WY, Zhang MM, Liu C, Kang Y, Wang JO, Yang XH (2019). Long noncoding RNA LINC00473 drives the progression of pancreatic cancer via upregulating programmed death-ligand 1 by sponging microRNA-195-5p. J Cell Physiol.

[102] Mao D, Hu C, Zhang J, Feng C, Zhang Z, Wang J (2019). Long Noncoding RNA GM16343 Promotes IL-36beta to Regulate Tumor Microenvironment by CD8(+)T cells. Technol Cancer Res Treat.

[103] Huang D, Chen J, Yang L, Ouyang Q, Li J, Lao L (2018). NKILA lncRNA promotes tumor immune evasion by sensitizing T cells to activation-induced cell death. Nat Immunol.

[104] Mineo M, Lyons SM, Zdioruk M, von Spreckelsen N, Ferrer-Luna R, Ito H (2020). Tumor Interferon Signaling Is Regulated by a lncRNA INCR1 Transcribed from the PD-L1 Locus. Mol Cell.

[105] Zhang RY, Ni F, Fu BQ, Wu Y, Sun R, Tian ZG (2016). A long noncoding RNA positively regulates CD56 in human natural killer cells. Oncotarget.

[106] Stein N, Berhani O, Schmiedel D, Duev-Cohen A, Seidel E, Kol I (2019). IFNG-AS1 Enhances Interferon Gamma Production in Human Natural Killer Cells. iScience.

[107] Wei MF, Gu ZS, Zheng LL, Zhao MX, Wang XJ (2020). Long non-coding RNA GAS5 promotes natural killer cell cytotoxicity against gastric cancer by regulating miR-18a. Neoplasma.

[108] Fang P, Xiang L, Chen W, Li S, Huang S, Li J (2019). LncRNA GAS5 enhanced the killing effect of NK cell on liver cancer through regulating miR-544/RUNX3. Innate Immun.

[109] Li S, Zhu A, Ren K, Li S, Chen L (2020). IFNbeta-induced exosomal linc-EPHA6-1 promotes cytotoxicity of NK cells by acting as a ceRNA for hsa-miR-4485-5p to up-regulate NKp46 expression. Life Sci.

[110] Tesi RJ (2019). MDSC; the Most Important Cell You Have Never Heard Of. Trends Pharmacol Sci.

[111] Gabrilovich D, Ishida T, Oyama T, Ran S, Kravtsov V, Nadaf S (1998). Vascular endothelial growth factor inhibits the development of dendritic cells and dramatically affects the differentiation of multiple hematopoietic lineages in vivo. Blood.

[112] Bronte V, Chappell DB, Apolloni E, Cabrelle A, Wang M, Hwu P (1999). Unopposed production of granulocyte-macrophage colony-stimulating factor by tumors inhibits CD8 + T cell responses by dysregulating antigen-presenting cell maturation. J Immunol.

[113] Gabrilovich DI, Bronte V, Chen SH, Colombo MP, Ochoa A, Ostrand-Rosenberg S (2007). The terminology issue for myeloid-derived suppressor cells. Cancer Res.

[114] Gabrilovich DI (2017). Myeloid-Derived Suppressor Cells. Cancer Immunol Res.

[115] Bronte V, Brandau S, Chen SH, Colombo MP, Frey AB, Greten TF (2016). Recommendations for myeloid-derived suppressor cell nomenclature and characterization standards. Nat Commun.

[116] Kumar V, Patel S, Tcyganov E, Gabrilovich DI (2016). The Nature of Myeloid-Derived Suppressor Cells in the Tumor Microenvironment. Trends Immunol.

[117] Haverkamp JM, Smith AM, Weinlich R, Dillon CP, Qualls JE, Neale G (2014). Myeloid-Derived Suppressor Activity Is Mediated by Monocytic Lineages Maintained by Continuous Inhibition of Extrinsic and Intrinsic Death Pathways. Immunity.

[118] Ugel S, De Sanctis F, Mandruzzato S, Bronte V (2015). Tumor-induced myeloid deviation: when myeloid-derived suppressor cells meet tumor-associated macrophages. J Clin Invest.

[119] Veglia F, Perego M, Gabrilovich D (2018). Myeloid-derived suppressor cells coming of age. Nat Immunol.

[120] Alkasalias T, Moyano-Galceran L, Arsenian-Henriksson M, Lehti K (2018). Fibroblasts in the Tumor Microenvironment: Shield or Spear?. Int J Mol Sci.

[121] Tartour E, Pere H, Maillere B, Terme M, Merillon N, Taieb J (2011). Angiogenesis and immunity: a bidirectional link potentially relevant for the monitoring of antiangiogenic therapy and the development of novel therapeutic combination with immunotherapy. Cancer Metastasis Rev.

[122] Shojaei F, Wu X, Qu X, Kowanetz M, Yu L, Tan M (2009). G-CSF-initiated myeloid cell mobilization and angiogenesis mediate tumor refractoriness to anti-VEGF therapy in mouse models. Proc Natl Acad Sci U S A.

[123] Zhang S, Ma X, Zhu C, Liu L, Wang G, Yuan X (2016). The Role of Myeloid-Derived Suppressor Cells in Patients with Solid Tumors: A Meta-Analysis. PLoS One.

[124] Thevenot PT, Sierra RA, Raber PL, Al-Khami AA, Trillo-Tinoco J, Zarreii P (2014). The stress-response sensor chop regulates the function and accumulation of myeloid-derived suppressor cells in tumors. Immunity.

[125] Ossipow V, Descombes P, Schibler U (1993). CCAAT/enhancer-binding protein mRNA is translated into multiple proteins with different transcription activation potentials. Proc Natl Acad Sci U S A..

[126] Marigo I, Bosio E, Solito S, Mesa C, Fernandez A, Dolcetti L (2010). Tumor-induced tolerance and immune suppression depend on the C/EBPbeta transcription factor. Immunity.

[127] Boulland ML, Marquet J, Molinier-Frenkel V, Moller P, Guiter C, Lasoudris F (2007). Human IL4I1 is a secreted L-phenylalanine oxidase expressed by mature dendritic cells that inhibits T-lymphocyte proliferation. Blood.

[128] Yue YP, Huang W, Liang JJ, Guo J, Ji J, Yao YL (2015). IL4I1 Is a Novel Regulator of M2 Macrophage Polarization That Can Inhibit T Cell Activation via L-Tryptophan and Arginine Depletion and IL-10 Production. Plos One.

[129] Hong EH, Chang SY, Lee BR, Kim YS, Lee JM, Kang CY (2013). Blockade of Myd88 signaling induces antitumor effects by skewing the immunosuppressive function of myeloid-derived suppressor cells. Int J Cancer.

[130] Delano MJ, Scumpia PO, Weinstein JS, Coco D, Nagaraj S, Kelly-Scumpia KM (2007). MyD88-dependent expansion of an immature GR-1(+)CD11b(+) population induces T cell suppression and Th2 polarization in sepsis. J Exp Med.

[131] Weber-Steffens D, Hunold K, Kurschner J, Martinez SG, Elumalai P, Schmidt D (2013). Immature mouse granulocytic myeloid cells are characterized by production of ficolin-B. Mol Immunol.

[132] Granot Z, Henke E, Comen EA, King TA, Norton L, Benezra R (2011). Tumor entrained neutrophils inhibit seeding in the premetastatic lung. Cancer Cell.

[133] Sionov RV, Fridlender ZG, Granot Z (2015). The Multifaceted Roles Neutrophils Play in the Tumor Microenvironment. Cancer Microenviron.

[134] Shaul ME, Fridlender ZG (2019). Tumour-associated neutrophils in patients with cancer. Nat Rev Clin Oncol.

[135] Galdiero MR, Varricchi G, Loffredo S, Mantovani A, Marone G (2018). Roles of neutrophils in cancer growth and progression. J Leukoc Biol.

[136] Coffelt SB, Wellenstein MD, de Visser KE (2016). Neutrophils in cancer: neutral no more. Nat Rev Cancer.

[137] Fridlender ZG, Sun J, Kim S, Kapoor V, Cheng G, Ling L (2009). Polarization of tumor-associated neutrophil phenotype by TGF-beta: “N1” versus “N2” TAN. Cancer Cell.

[138] Giese MA, Hind LE, Huttenlocher A (2019). Neutrophil plasticity in the tumor microenvironment. Blood.

[139] Ruffell B, Affara NI, Coussens LM (2012). Differential macrophage programming in the tumor microenvironment. Trends Immunol.

[140] de Oliveira S, Rosowski EE, Huttenlocher A (2016). Neutrophil migration in infection and wound repair: going forward in reverse. Nat Rev Immunol.

[141] Gardner A, Ruffell B (2016). Dendritic Cells and Cancer Immunity. Trends Immunol.

[142] Giovanelli P, Sandoval TA, Cubillos-Ruiz JR (2019). Dendritic Cell Metabolism and Function in Tumors. Trends Immunol.

[143] Nefedova Y, Huang M, Kusmartsev S, Bhattacharya R, Cheng PY, Salup R (2004). Hyperactivation of STAT3 is involved in abnormal differentiation of dendritic cells in cancer. J Immunol.

[144] Wang SS, Liu W, Ly D, Xu H, Qu LM, Zhang L (2019). Tumor-infiltrating B cells: their role and application in anti-tumor immunity in lung cancer. Cell Mol Immunol.

[145] Yasuda M, Mizukami M, Hanagiri T, Shigematsu Y, Fukuyama T, Nagata Y (2006). Antigens recognized by IgG derived from tumor-infiltrating B lymphocytes in human lung cancer. Anticancer Res.

[146] Zirakzadeh AA, Marits P, Sherif A, Winqvist O (2013). Multiplex B cell characterization in blood, lymph nodes, and tumors from patients with malignancies. J Immunol.

[147] Maletzki C, Jahnke A, Ostwald C, Klar E, Prall F, Linnebacher M (2012). Ex-vivo clonally expanded B lymphocytes infiltrating colorectal carcinoma are of mature immunophenotype and produce functional IgG. PLoS One.

[148] Sarvaria A, Madrigal JA, Saudemont A (2017). B cell regulation in cancer and anti-tumor immunity. Cell Mol Immunol.

[149] Zhang Y, Gallastegui N, Rosenblatt JD (2015). Regulatory B cells in anti-tumor immunity. Int Immunol.

[150] Klinker MW, Lundy SK (2012). Multiple mechanisms of immune suppression by B lymphocytes. Mol Med.

[151] Verma A, Jiang Y, Du W, Fairchild L, Melnick A, Elemento O (2015). Transcriptome sequencing reveals thousands of novel long non-coding RNAs in B cell lymphoma. Genome Med.

[152] Boxer LM, Dang CV (2001). Translocations involving c-myc and c-myc function. Oncogene.

[153] Su Y, Sun B, Lin X, Zhao X, Ji W, He M (2016). Therapeutic strategy with artificially-designed i-lncRNA targeting multiple oncogenic microRNAs exhibits effective antitumor activity in diffuse large B-cell lymphoma. Oncotarget.

[154] Raimondi G, Turner MS, Thomson AW, Morel PA (2007). Naturally occurring regulatory T cells: recent insights in health and disease. Crit Rev Immunol.

[155] Roncarolo MG, Gregori S, Battaglia M, Bacchetta R, Fleischhauer K, Levings MK (2006). Interleukin-10-secreting type 1 regulatory T cells in rodents and humans. Immunol Rev.

[156] Wang Y, Su MA, Wan YY (2011). An essential role of the transcription factor GATA-3 for the function of regulatory T cells. Immunity.

[157] Carvalho MI, Pires I, Prada J, Gregorio H, Lobo L, Queiroga FL (2016). Intratumoral FoxP3 expression is associated with angiogenesis and prognosis in malignant canine mammary tumors. Vet Immunol Immunop.

[158] Ricciuti B, Foglietta J, Bianconi V, Sahebkar A, Pirro M (2019). Enzymes involved in tumor-driven angiogenesis: A valuable target for anticancer therapy. Semin Cancer Biol.

[159] Buckner JH (2010). Mechanisms of impaired regulation by CD4(+)CD25(+)FOXP3(+) regulatory T cells in human autoimmune diseases. Nat Rev Immunol.

[160] Dannull J, Su Z, Rizzieri D, Yang BK, Coleman D, Yancey D (2005). Enhancement of vaccine-mediated antitumor immunity in cancer patients after depletion of regulatory T cells. J Clin Invest.

[161] Bates GJ, Fox SB, Han C, Leek RD, Garcia JF, Harris AL (2006). Quantification of regulatory T cells enables the identification of high-risk breast cancer patients and those at risk of late relapse. J Clin Oncol.

[162] Perrone G, Ruffini PA, Catalano V, Spino C, Santini D, Muretto P (2008). Intratumoural FOXP3-positive regulatory T cells are associated with adverse prognosis in radically resected gastric cancer. Eur J Cancer.

[163] Bohling SD, Allison KH (2008). Immunosuppressive regulatory T cells are associated with aggressive breast cancer phenotypes: a potential therapeutic target. Mod Pathol.

[164] Xia M, Liu J, Liu S, Chen K, Lin H, Jiang M (2017). Ash1l and lnc-Smad3 coordinate Smad3 locus accessibility to modulate iTreg polarization and T cell autoimmunity. Nat Commun.

[165] Zemmour D, Pratama A, Loughhead SM, Mathis D, Benoist C (2017). Flicr, a long noncoding RNA, modulates Foxp3 expression and autoimmunity. Proc Natl Acad Sci U S A.

[166] Brajic A, Franckaert D, Burton O, Bornschein S, Calvanese AL, Demeyer S (2018). The Long Non-coding RNA Flatr Anticipates Foxp3 Expression in Regulatory T Cells. Front Immunol.

[167] Hudson WH, Prokhnevska N, Gensheimer J, Akondy R, McGuire DJ, Ahmed R (2019). Expression of novel long noncoding RNAs defines virus-specific effector and memory CD8(+) T cells. Nat Commun.

[168] Pang KC, Dinger ME, Mercer TR, Malquori L, Grimmond SM, Chen W (2009). Genome-wide identification of long noncoding RNAs in CD8 + T cells. J Immunol.

[169] Saadi W, Kermezli Y, Dao LTM, Mathieu E, Santiago-Algarra D, Manosalva I (2019). A critical regulator of Bcl2 revealed by systematic transcript discovery of lncRNAs associated with T-cell differentiation. Sci Rep.

[170] Kotzin JJ, Iseka F, Wright J, Basavappa MG, Clark ML, Ali MA (2019). The long noncoding RNA Morrbid regulates CD8 T cells in response to viral infection. Proc Natl Acad Sci U S A.

[171] Wu J, Niu Q, Yuan J, Xu X, Cao L (2020). lncRNA-CD160 decreases the immunity of CD8(+) T cells through epigenetic mechanisms in hepatitis B virus infection. Oncol Lett.

[172] Wang X, Shen H, He Q, Tian W, Xia A, Lu XJ (2019). Exosomes derived from exhausted CD8 + T cells impaired the anticancer function of normal CD8 + T cells. J Med Genet.

[173] Chiossone L, Dumas PY, Vienne M, Vivier E (2018). Natural killer cells and other innate lymphoid cells in cancer. Nat Rev Immunol.

[174] Shimasaki N, Jain A, Campana D (2020). NK cells for cancer immunotherapy. Nat Rev Drug Discov.

[175] Malmberg KJ, Carlsten M, Bjorklund A, Sohlberg E, Bryceson YT, Ljunggren HG (2017). Natural killer cell-mediated immunosurveillance of human cancer. Semin Immunol.

[176] Pasero C, Gravis G, Granjeaud S, Guerin M, Thomassin-Piana J, Rocchi P (2015). Highly effective NK cells are associated with good prognosis in patients with metastatic prostate cancer. Oncotarget.

[177] Souza-Fonseca-Guimaraes F (2016). NK cell-based immunotherapies: awakening the innate anti-cancer response. Discov Med.

[178] Mace EM, Gunesch JT, Dixon A, Orange JS (2016). Human NK cell development requires CD56-mediated motility and formation of the developmental synapse. Nat Commun.

[179] Bhan A, Soleimani M, Mandal SS (2017). Long Noncoding RNA and Cancer: A New Paradigm. Can Res.

[180] Jiang C, Li X, Zhao H, Liu H (2016). Long non-coding RNAs: potential new biomarkers for predicting tumor invasion and metastasis. Mol Cancer.

[181] Qi P, Zhou XY, Du X (2016). Circulating long non-coding RNAs in cancer: current status and future perspectives. Mol Cancer.

[182] Gutschner T, Baas M, Diederichs S (2011). Noncoding RNA gene silencing through genomic integration of RNA destabilizing elements using zinc finger nucleases. Genome Res.

[183] Ozcan G, Ozpolat B, Coleman RL, Sood AK, Lopez-Berestein G (2015). Preclinical and clinical development of siRNA-based therapeutics. Adv Drug Deliver Rev.

[184] Arun G, Diermeier S, Akerman M, Chang KC, Wilkinson JE, Hearn S (2016). Differentiation of mammary tumors and reduction in metastasis upon Malat1 lncRNA loss. Gene Dev.

[185] Pavco PA, Bouhana KS, Gallegos AM, Agrawal A, Blanchard KS, Grimm SL (2000). Antitumor and antimetastatic activity of ribozymes targeting the messenger RNA of vascular endothelial growth factor receptors. Clin Cancer Res.

[186] Hu Q, Egranov SD, Lin C, Yang L (2020). Long noncoding RNA loss in immune suppression in cancer. Pharmacol Ther.

[187] Kampmann M (2018). CRISPRi and CRISPRa Screens in Mammalian Cells for Precision Biology and Medicine. ACS Chem Biol.

[188] Mizrahi A, Czerniak A, Levy T, Amiur S, Gallula J, Matouk I (2009). Development of targeted therapy for ovarian cancer mediated by a plasmid expressing diphtheria toxin under the control of H19 regulatory sequences. J Transl Med.

[189] Grupp SA, Kalos M, Barrett D, Aplenc R, Porter DL, Rheingold SR (2013). Chimeric Antigen Receptor-Modified T Cells for Acute Lymphoid Leukemia. New Engl J Med.

[190] Maude SL, Frey N, Shaw PA, Aplenc R, Barrett DM, Bunin NJ (2014). Chimeric Antigen Receptor T Cells for Sustained Remissions in Leukemia. New Engl J Med.

[191] Vaidya AM, Sun Z, Ayat N, Schilb A, Liu X, Jiang H (2019). Systemic Delivery of Tumor-Targeting siRNA Nanoparticles against an Oncogenic LncRNA Facilitates Effective Triple-Negative Breast Cancer Therapy. Bioconjug Chem.

[192] Fan Q, Yang L, Zhang X, Peng X, Wei S, Su D (2018). The emerging role of exosome-derived non-coding RNAs in cancer biology. Cancer Lett.

